# Supplementary material for: After the honeymoon, the divorce: Unexpected outcomes of disease control measures against endemic infections
Source: PLoS Comput Biol. 2020 Oct 19;16(10):e1008292. doi: 10.1371/journal.pcbi.1008292 (PMC7595641; doi:10.1371/journal.pcbi.1008292)
Supplement: S1 Text — Includes results from host-vector, seasonal host-vector, SIRS, HIV, and age-structured models, the analytic approximation, and additional sensitivity analysis. (PDF) [file pcbi.1008292.s001.pdf]

Supplementary Information for:

Title: After the honeymoon, the divorce: unexpected outcomes of disease control measures against endemic infections

Short Title: Unexpected outcomes of disease control measures against endemic infections

Authors: Brandon Hollingsworth[a], Kenichi W Okamoto[b], Alun L Lloyd[a,c]

Corresponding Author: Brandon Hollingsworth

Affiliations:

a) Biomathematics Graduate Program, North Carolina State University, Raleigh, NC 27695, USA
b) Department of Biology, University of St. Thomas, St. Paul, MN, 55105, USA
c) Department of Mathematics, North Carolina State University, Raleigh, NC 27695, USA

Keywords: Disease Modeling, Endemic Disease, Control Cessation

Additional Models:

SIR Model with Changing Population Size

We assume a population similar to the SIR model in the main text, with the exception that the per capita birth and death rate are allowed to differ. We have

$$\begin{aligned} \dot{S} &= bN - \mu S - \beta \frac{S(I + I_b)}{N} \\ \dot{I} &= \beta \frac{S(I + I_b)}{N} - (\gamma + \mu)I \\ \dot{N} &= (b - \mu)N. \end{aligned} \tag{S1}$$

Here b is the per capita birth rate and μ is the per capita death rate. For illustration, we take two values of b, $b = 1.25\mu$ and $b = .75\mu$, corresponding to 25% population growth or reduction per year. While this is an extreme case, we expect that any effect on the magnitude of the divorce effect would most likely be seen in the extremes. Since the endemic equilibrium is not well defined for a changing population, we simulate the population for a thousand years before starting control. Initial values of N were chosen so that at the end of the thousand years, the population size was 1×10^6. We see that the growth (Figure S16a), or decline (Figure S16b), of the population does not eliminate the divorce effect, but does affect the magnitude and timing of the post-control outbreak, with a larger and earlier post-control outbreak in the growing population due to a larger number of susceptible individuals being born.

SIR Model with Vaccination

To model vaccination against infection, we assume that some portion, v, of births enter the recovered class instead of the susceptible class, while all other dynamics proceed similarly to the SIR model (Equations S2). For illustration, we take $v = .5$ and assume the vaccination campaign lasts one year before being discontinued. We see that during the control period the proportion of the population that is infective falls significantly more slowly than with transmission reduction (Figure S17). Following the end of control, we see a series of post-control outbreaks that bring the infective proportion of the population above endemic levels, but they are not large enough to bring RCI above 1. This lack of divorce effect is due directly to the maintenance of population level immunity due to the vaccination, which keeps the susceptible population from being able to build sufficiently. It is important to note that, as shown in Okamoto et al. [1], it is possible to see the divorce effect in combined controls that involve both immunizing and non-immunizing controls.

$$\begin{aligned} \dot{S} &= b(1 - v)(N - S) - \beta \frac{S(I + I_b)}{N} \\ \dot{I} &= \beta \frac{S(I + I_b)}{N} - (\gamma + \mu)I \end{aligned} \tag{S2}$$

SIRS Model

We assume a well-mixed population with parameters defined as in the main text. However, instead of permanent immunity, we assume that immunity is lost at per-capita rate l, such that the average length of immunity following an infection is $1/l$ (Equation S3). For the sake of illustration, $l = 1/10\ \text{year}^{-1}$, corresponding to an average of 10 years of immunity following recovery.

$$\begin{aligned} \dot{S} &= \mu(N-S) - \beta\frac{S(I+I_b)}{N} + lR \\ \dot{I} &= \beta\frac{S(I+I_b)}{N} - (\gamma + \mu)I \\ \dot{R} &= \gamma I - (\mu + l)R \end{aligned} \qquad \text{(S3)}$$

Similar to the SIR model, we see suppression of the infection for a period of time during and immediately following the control (Figure S18). A large post-control outbreak is seen about 3 months after the end of treatment. This outbreak is sufficiently large to bring the RCI above 1, to about 1.45, before the outbreak subsides and prevalence and RCI fall again. As the immune period following infection shrinks towards zero, the SIRS model approaches the behavior of an SIS model. This results in the magnitude of the divorce effect being reduced as the immune period, and the population of immune individuals, becomes smaller.

Within-Host Virus Dynamics (HIV) Model

We examine the divorce effect in the model for the within-host dynamics of HIV presented in Rong and Perelson [2], with all equations and parameters taken directly from their text (Equations S4 and Table S1). Here, *T* stands for the concentration of target cells, *L* for latently infected cells, *T** for actively infected cells, V_I for infectious virions, and V_{NI} for non-infectious (defective) virions. Parameter names and values are given in Table 1. Here, cumulative incidence is in terms of actively infectious T cells. We see that the divorce effect does occur following a 25 day treatment that has both a protease inhibitor and reverse transcriptase inhibitor with efficacies of 50% (Figure S19).

$$\begin{aligned} \dot{T} &= \lambda - d_T T - (1-\epsilon_{RT})kV_I T \\ \dot{L} &= \alpha_L(1-\epsilon_{RT})kV_I T - d_L L - aL \\ \dot{T^*} &= (1-\alpha_L)(1-\epsilon_{RT}) - \delta T^* + \alpha L \\ \dot{V_I} &= (1-\epsilon_{PI})N\delta T^* - cV_I \\ \dot{V_{NI}} &= \epsilon_{PI}N\delta T^* - cV_{NI} \end{aligned} \qquad \text{(S4)}$$

Age-structured Model with Realistic Mixing

Here we show the presence of the divorce effect in an age-structured model with realistic mixing between groups. This model, and code, is from a tutorial given by Aaron King and Helen Wearing [3]. We assume that there 30 age-groups, with ages 0-19 occurring as single year age groups, 20-75 as 5 year age groups. Transitions between compartments occur according to Equation S5, note that we use ∘ to denote elementwise multiplication. In which *A* is a matrix describing transitions between age classes, e.g. aging and deaths, *b* is a matrix describing births with a constant birth rate as its first element and zeros everywhere else. New-born susceptibles

enter the youngest age class at a rate of *b* = 100/year, movement between the age classes takes on average 1 year for ages 0-20, 5 years for ages 21-75, and death occurs at a constant rate in the last age class, occurring on average after 15 years. S, I, and R are vectors containing the numbers of individuals of each age class that are susceptible, infective, or immune, respectively. β is a matrix containing the transmission parameters for infection occurring within and between age classes, and is constructed by taking a matrix of age-specific contact rates and multiplying it by a constant rate of infection per contact. This contact network is based on [4] and freely available online, and the constant rate of infection per contact chosen so that $R_0 = 5$. γ is a vector containing the rate of recovery of individuals in each age class, but is assumed to be constant across all age classes and is the same as the main text ($\gamma = 73$ /year). Control works, as in the SIR model, by reducing the transmission parameter by 50% and lasts one year.

$$\begin{aligned} \dot{S} &= -\beta I \circ S + AS + \mathrm{b} \\ \dot{I} &= \beta I \circ S + AI - \gamma I \\ \dot{R} &= AR + \gamma I \end{aligned} \quad \text{(S5)}$$

We see that, similar to the non-structured SIR model, there is a period of time, lasting about 4 years, in which RCI is falling, before a large outbreak brings RCI above 1 (Figure S20). Importantly, while the magnitude of the effect varies across groups, due to mixing, its presence does not.

Analytical Approximation:

Here we describe a crude analytical approximation for the magnitude of the divorce effect in the simplest setting of a non-seasonal directly transmitted infection (i.e. the SIR model), and based on the well-known analysis of the size of an outbreak in a closed population [5,6]. We assume that the post-control outbreak occurs immediately following the end of the control period and that the outbreak happens instantaneously. Further, we assume that control is perfect, so that there are no new cases of infection during the control period, and that all individuals that are infective before the control begins recover by the end of the control period. When control begins, the population can be subdivided into individuals that are susceptible and those that have previously been exposed and will be immune when the control is ended. Assuming $R_0>1$, *t*he numbers in these two groups are determined by the endemic equilibrium, where $S^* = N/R_0$ and $R^* = N(1 - 1/R_0)$. The number in the latter group decays exponentially due to mortality and the number of susceptibles grows at the same rate because of births (noting that the population size is taken to be constant). This gives the number of susceptible individuals at the time control ends, t_{end}, as

$$S = N\left(\frac{1}{R_0} + \left(1 - \frac{1}{R_0}\right)(1 - e^{-\mu t_{end}})\right) \quad \text{(S6)}$$

Once the control is ended, the infection is assumed to be reintroduced immediately by a small number of infectious individuals and occurs instantaneously, meaning that demography does not affect the final outbreak size. This means that the post-control outbreak size, *Z*, can be found by solving the familiar transcendental equation:

$$Z = S\left(1 - e^{-R_0\left(\frac{Z}{N}\right)}\right). \quad \text{(S7)}$$

The post-control outbreak size is then compared to the cumulative number of infections that would be expected in the endemic case to find the predicted RCI (Equation S8).

$$\mathrm{RCI} = \frac{Z}{\mu N\left(1 - \frac{1}{R_0}\right)t} \quad \text{(S8)}$$

Results of Analytical Approximation

When compared to the simulations, our analytical approximation overestimates the magnitude of the divorce effect (Figure S21(a)). This is in direct contrast to simulations where the outbreak requires a long accumulation of infectives, often happens years later, and takes some time to occur. This approximation performs best in the most biologically relevant portion of parameter space (R_0<10 and control lasting less than 20 years), where the error is generally below 20% (Figure S21(b)), however it performs very poorly for extremely short durations of control.

Sensitivity to Background Force of Infection

Deterministic compartmental epidemiological models suffer from the well-known weakness that the numbers of infectives can fall to arbitrarily low levels. To combat this, a background force of infection is often included in such models, representing infections due to contact with populations outside the focal population [7]. In our model, this process is accounted for by adding I_b to the number of infectives in the transmission term. The background force of infection, which is taken to be small compared to the within-patch force of infection at the endemic state, ensures that there is a low level of transmission in the population, even as the number of infectives falls during the control period, and acts to reseed infection following control. In doing this, the background force of infection controls how quickly an outbreak will occur following the end of control, and hence can play an important role in determining the magnitude of the divorce effect. In general, a lower background force of infection means a later post-control outbreak, and often a larger divorce effect, while a higher background force of infection means an earlier post-control outbreak, less time for the build-up of the susceptible population, and a smaller divorce effect. These effects are most noticeable for a short-lived control. At a sufficient level, the background force of infection is large enough to drive the overall dynamics of the system, eliminating the divorce effect. When this occurs, the dynamics become driven by exogenous factors, similar to a sylvatic infection, reducing the importance of local infections. In addition to affecting the magnitude of the divorce effect, increasing I_b increases the rate at which the system approaches its endemic equilibrium following the end of control. This results in subsequent outbreaks being increasingly diminished. For our manuscript, we choose to use a realistic value of $I_b = 1$ for our models, compared to an endemic level of 183 infective individuals for these parameter values in the nonseasonal model. Figure S22 shows that for values of I_b that are sufficiently large to eliminate the divorce effect would require I_b to be roughly the same size as the endemic infection level.

It is well known that seasonally forced models are even more prone to having their numbers of infectives falling to low levels between outbreaks, with a background force of infection being commonly employed to counter this effect. Stronger seasonality magnifies this effect. Hence the background force of infection impacts the magnitude of the divorce effect, and given that the timing of control plays an important role in seasonal settings, there is an interaction between seasonality, the timing of the control, and the background force of infection in such cases. In general, as seasonality increases so does the difference between the maximum and minimum prevalence levels in the population. This results in an interaction between the background force of infection, the magnitude of seasonality, and the timing of the control determining the final magnitude of the divorce effect (Figures S8 and S23). This is important for predicting the magnitude of the divorce effect in real world situations, as there is a large amount of uncertainty associated with estimates of all three of these parameters. Importantly, below a specific background force of infection, the divorce effect is seen for all values of these parameters.

Citations

1. Okamoto KW, Gould F, Lloyd AL. 2016 Integrating Transgenic Vector Manipulation with Clinical Interventions to Manage Vector-Borne Diseases. *PLOS Comput. Biol.* **12**, e1004695. (doi:10.1371/journal.pcbi.1004695)
2. Rong L, Perelson AS. 2009 Asymmetric division of activated latently infected cells may explain the decay kinetics of the HIV-1 latent reservoir and intermittent viral blips. *Math. Biosci.* **217**, 77–87. (doi:https://doi.org/10.1016/j.mbs.2008.10.006)
3. King AA, Wearing HJ. 2011 Age Structured Models.
4. Mossong J *et al.* 2008 Social contacts and mixing patterns relevant to the spread of infectious diseases. *PLoS Med* **5**. (doi:10.1371/journal.pmed.0050074)
5. Ma J, Earn DJD. 2006 Generality of the Final Size Formula for an Epidemic of a Newly Invading Infectious Disease. *Bull. Math. Biol.* **68**, 679–702. (doi:10.1007/s11538-005-9047-7)
6. Diekmann O, Heesterbeek JAP. 2000 *Mathematical Epidemiology of Infectious Diseases: Model Building, Analysis and Interpretation - O. Diekmann, J. A. P. Heesterbeek*.
7. Ferguson NM, Nokes DJ, Anderson RM. 1996 Dynamical complexity in age-structured models of the transmission of the measles virus: Epidemiological implications at high levels of vaccine uptake. *Math. Biosci.* (doi:10.1016/S0025-5564(96)00127-7)
