# Supplementary figures and images for: After the honeymoon, the divorce: Unexpected outcomes of disease control measures against endemic infections
Source: PLoS Comput Biol. 2020 Oct 19;16(10):e1008292. doi: 10.1371/journal.pcbi.1008292 (PMC7595641; doi:10.1371/journal.pcbi.1008292)

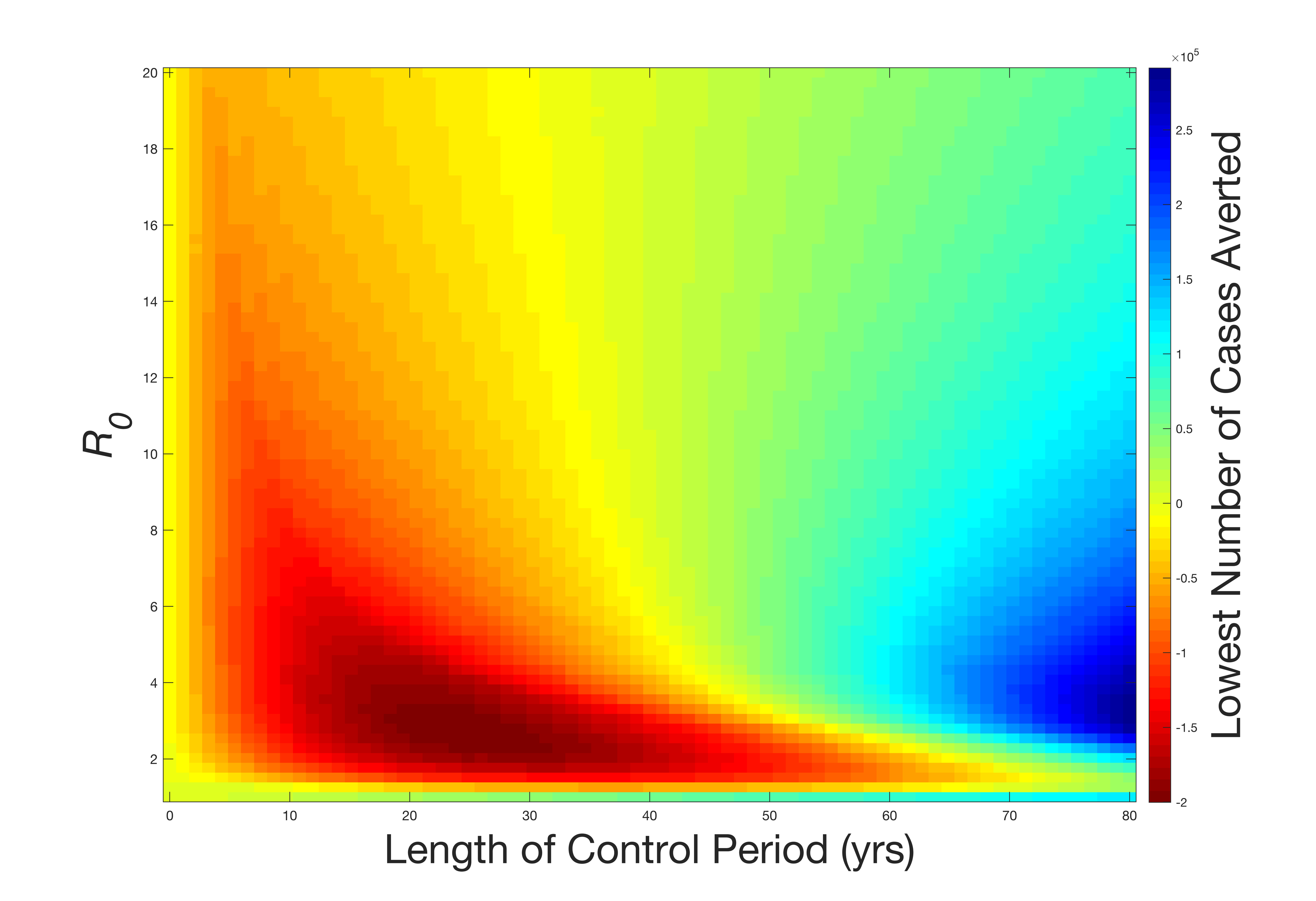

Supplement: S1 Fig — All parameters the same as in Fig 1(b). When measuring the success of a control program in terms of cases averted as opposed to RCI, the overall results are retained, with negative values of cases averted corresponding to an RCI > 1. Parameters are as in Fig 1(b) for comparison. (TIF) [file pcbi.1008292.s003.tif]

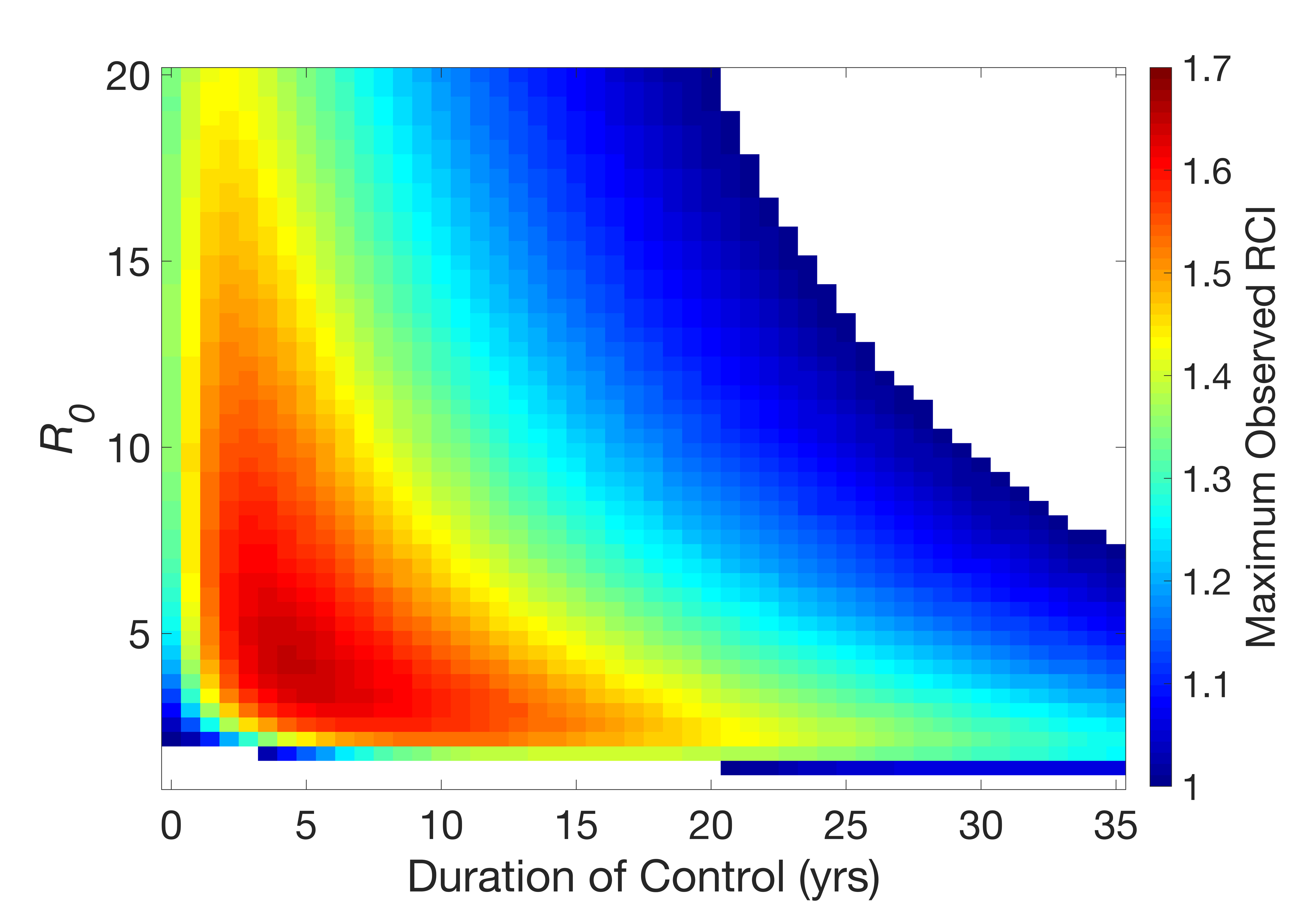

Supplement: S2 Fig — All parameters are as in Fig 1(b). The divorce effect is still observed if the control increases the recovery rate, γ, as opposed to decreasing the transmission parameter. All parameters the same as in Fig 1. Control increases γ to 730 (previously 73 /year). (TIF) [file pcbi.1008292.s004.tif]

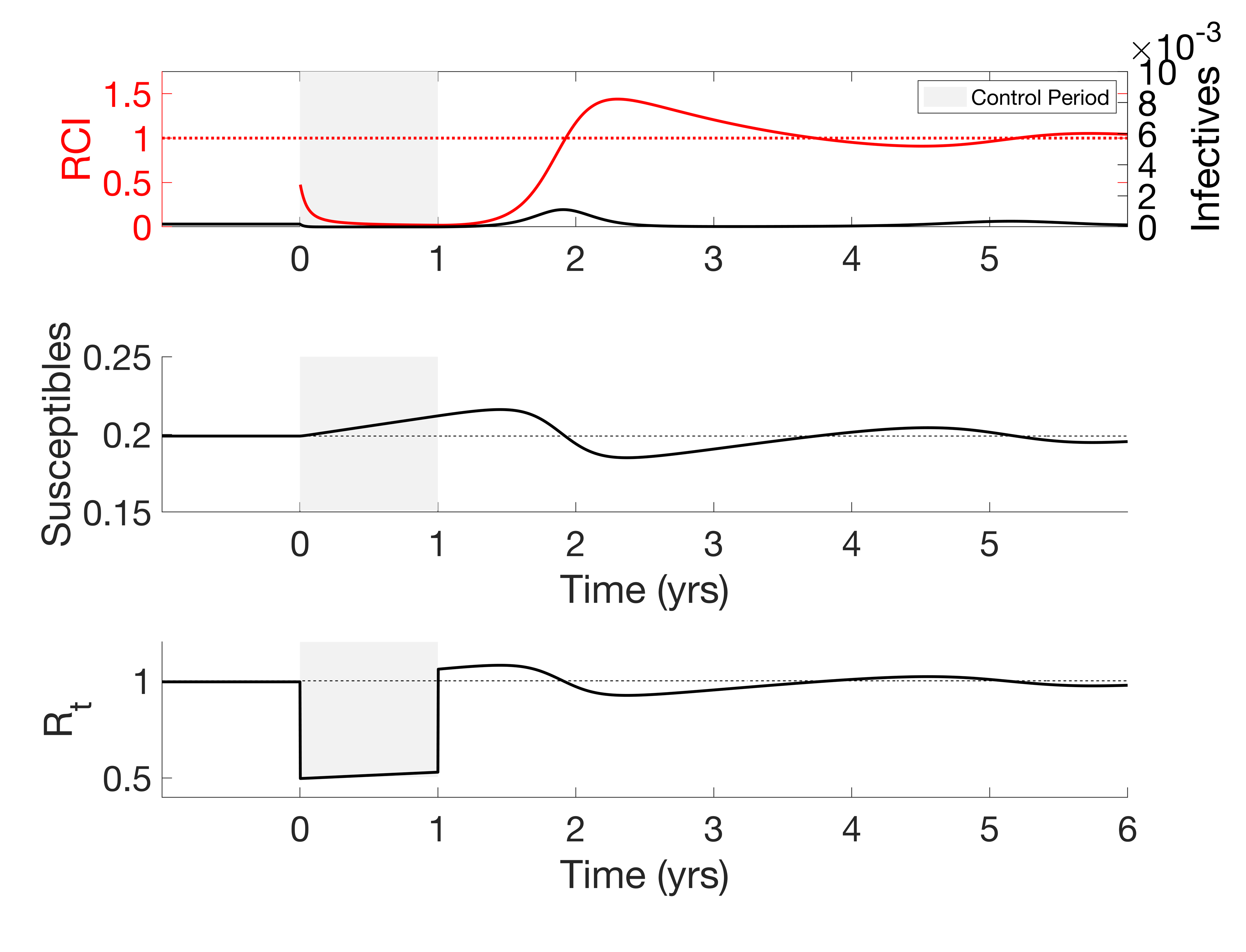

Supplement: S3 Fig — Figure corresponds to Fig 1(a) of the main text. Beginning at time zero, a year-long 50% reduction in the transmission parameter of an endemic infection (R0 = 5) reduces prevalence of the infection to near zero for the length of the control, where it remains until time 1.5 yrs, at which point a large post-control outbreak occurs. RCI falls towards zero as prevalence remains low, but the post-control outbreak is large enough to bring RCI well above 1 (approx. 1.6). Panel 2 shows that the susceptible population begins to rise during the control period and continues until the outbreak depletes the susceptible population. Likewise, the reproductive number at time t, Rt, begins to rise during the control period. Once the control is released and the transmission rate retains its original value, Rt increases above one and continues to grow until an outbreak occurs. (TIF) [file pcbi.1008292.s005.tif]

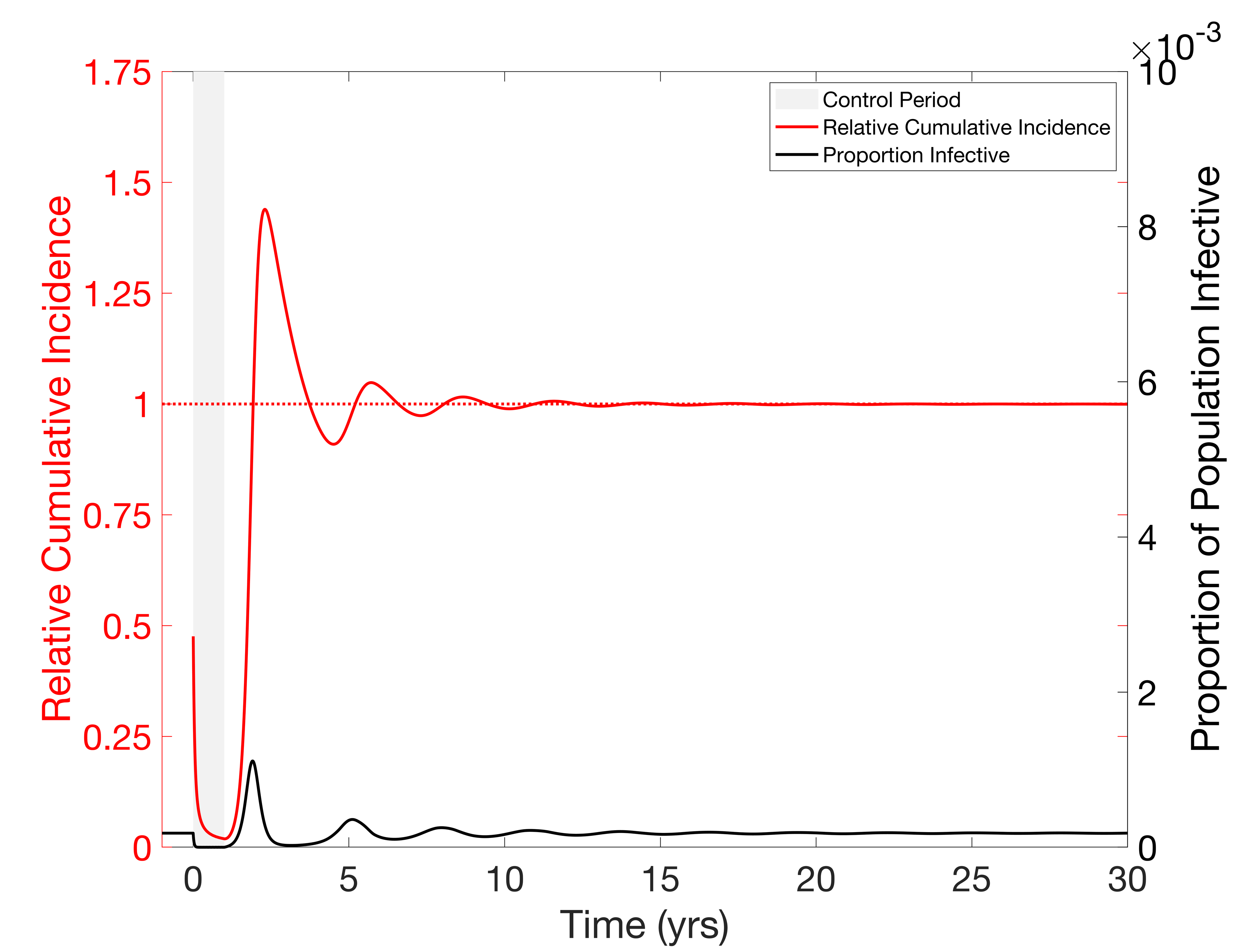

Supplement: S4 Fig — Figure corresponds to Fig 1(a) of the main text. Following a one year control in which the transmission parameter is reduced by 50%, the host population continues to experience outbreaks that bring RCI above one until the infection approaches the endemic state and RCI approaches one. (TIF) [file pcbi.1008292.s006.tif]

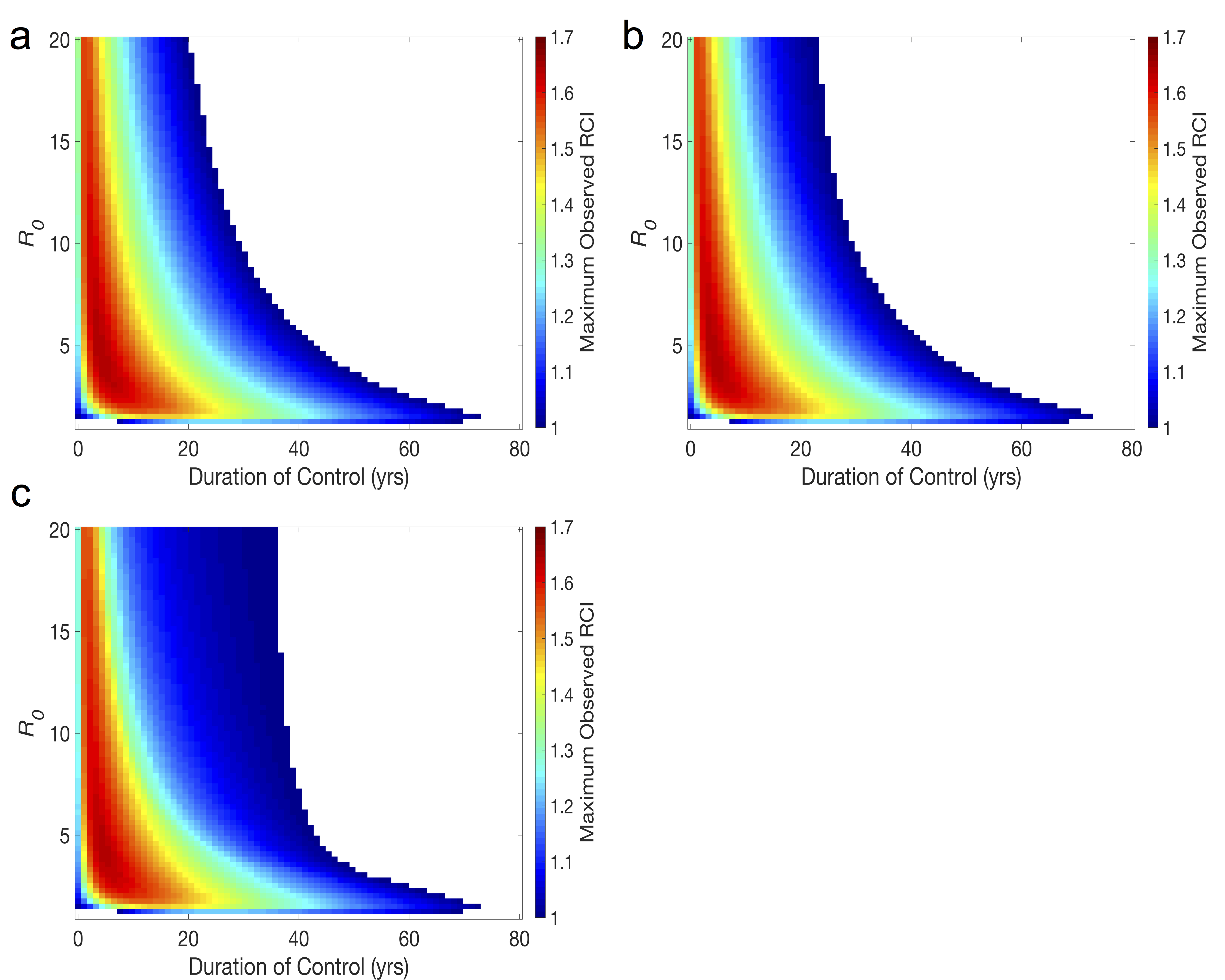

Supplement: S5 Fig — We see that the divorce effect occurs in a significant area of the parameter space with controls that reduce the transmission parameter, β, by 100% (a), 75% (b), and 50% (c). In each case, the only way to avoid the divorce effect is to maintain control for more than 20 years (or approximately 40 years in the case of 50% control). (TIF) [file pcbi.1008292.s007.tif]

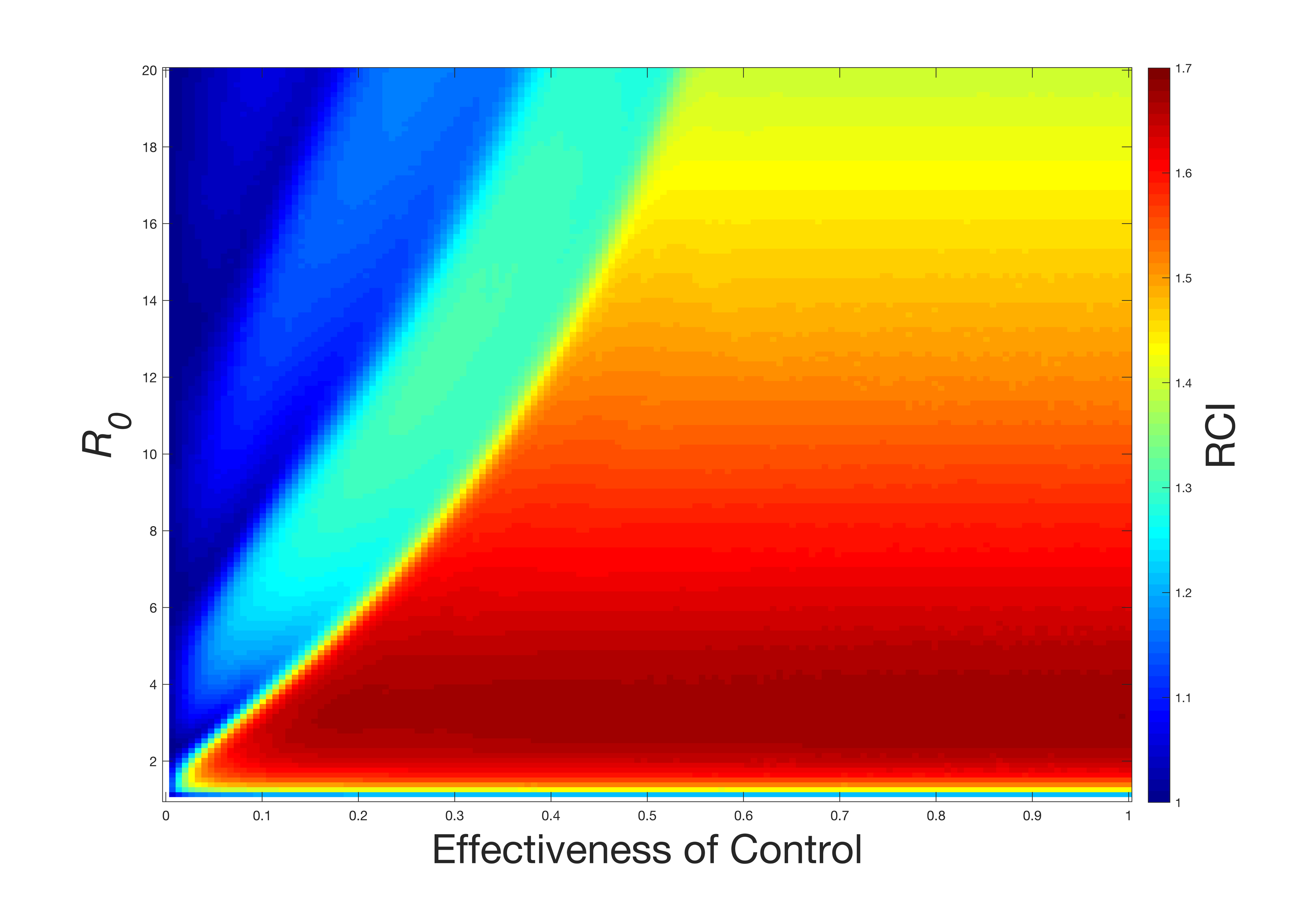

Supplement: S6 Fig — All controls are assumed to last for 1 year. The maximum RCI is found as the maximum observed RCI within 25 yrs after the end of a control that is between 0% and 100% effective for an infection with an R0 of between 0 and 20. The areas of lowered maximum RCI result from outbreaks due to honeymoon effect outbreaks deplenishing the population of susceptible individuals before the control periods end. (TIF) [file pcbi.1008292.s008.tif]

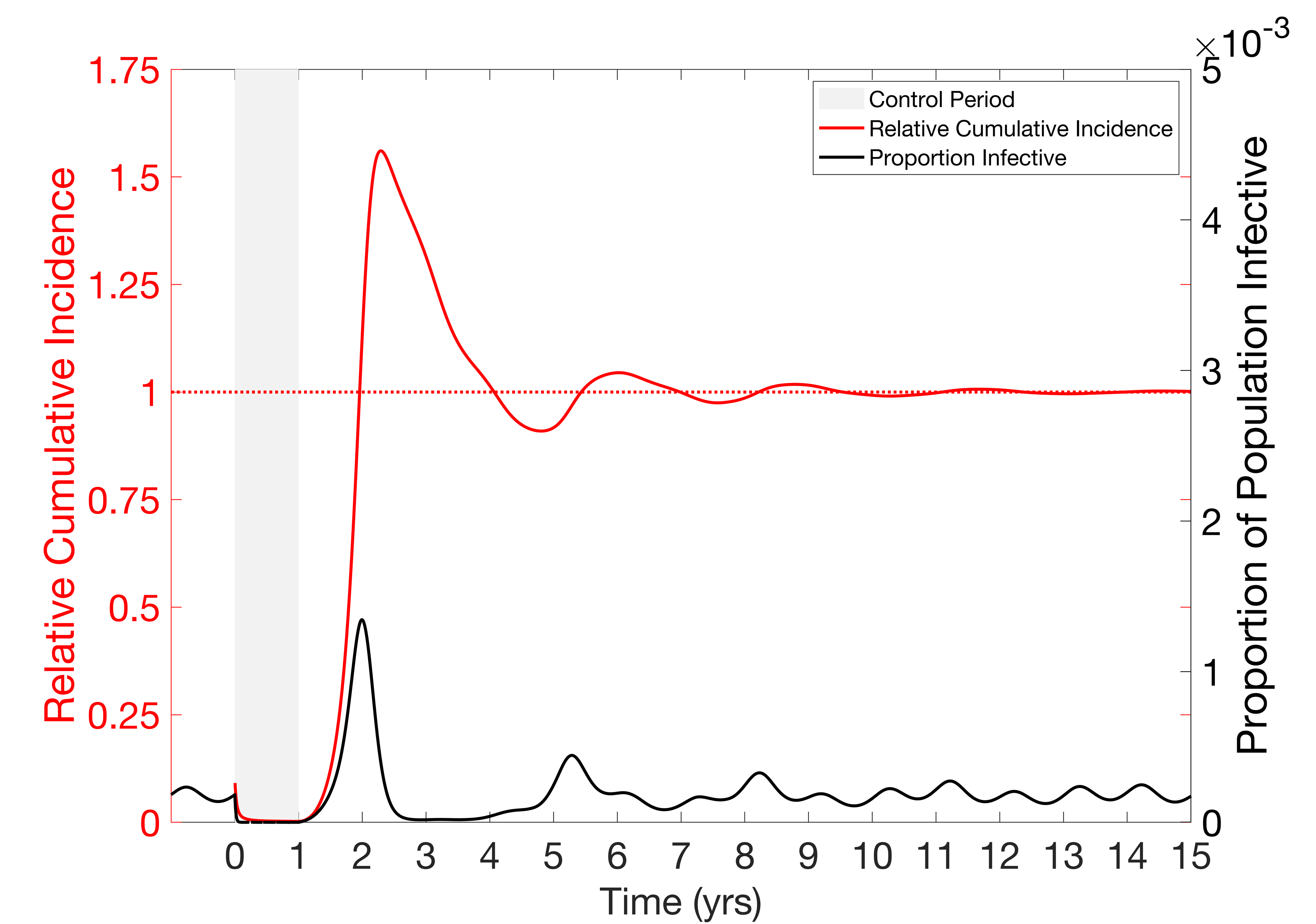

Supplement: S7 Fig — Following a one year control in which the transmission parameter is reduced by 50%, the host population continues to experience outbreaks that bring RCI above one until the infection approaches the endemic state and RCI approaches one. (TIF) [file pcbi.1008292.s009.tif]

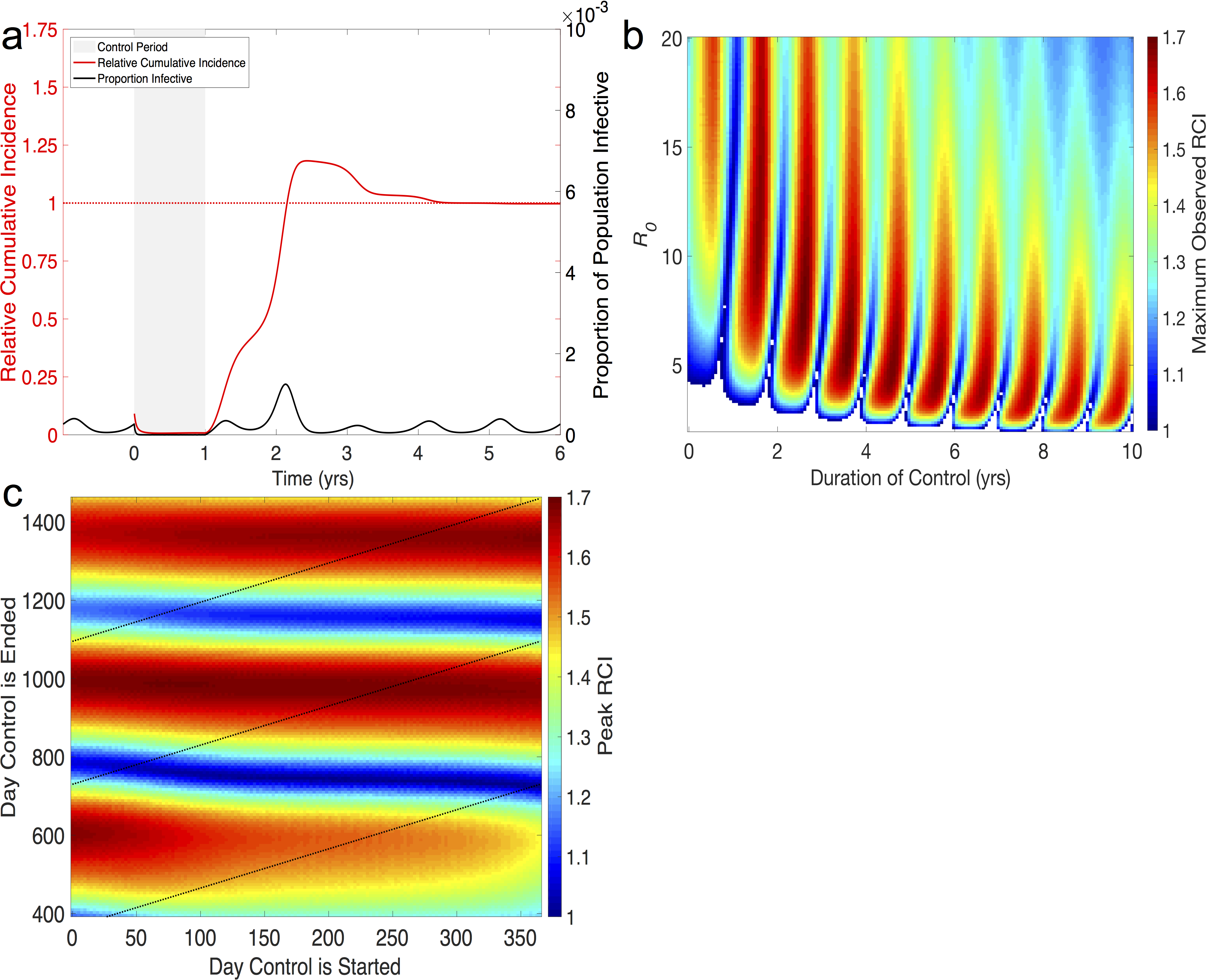

Supplement: S8 Fig — β1 = .1, Ib = 10., all other parameters as in Fig 2. Here a higher value of Ib is taken to adjust for the higher seasonality of the model. (a) Typical time-series showing the divorce effect. Beginning at time zero, when the transmission parameter is at its maximum, a year-long 90% reduction in the transmission rate of an endemic infection (R0 = 5) is implemented at the beginning of a seasonal outbreak and reduces prevalence of the infection to near zero for the length of the control. Following the end of the control, a small late season outbreak occurs, followed by a large outbreak during the next season. RCI falls towards zero as prevalence remains low while the control is in effect, rises slightly during the small late season outbreak, and rises above 1 during the large outbreak the following year. (b) Magnitude of divorce effect in terms of relative cumulative incidence (RCI). Maximum RCI is found as the highest value RCI observed within 25 yrs following a 100% effective control of an infection with 1<R0<20 and lasting between 1 month and 20 years. RCI>1 indicates the divorce effect and we see that divorce effect occurs in most of the parameter space. Unlike the SIR model (Fig 1), the magnitude of the divorce effect is not solely dependent on R0. (c) Effect of timing on the magnitude of the divorce effect. Maximum RCI is the highest RCI observed within 25 yrs following a 100% effective control of an infection with R0 = 10 beginning and ending on specified days. Dashed lines represent controls lasting either 1, 2, or 3 years. Maximum RCI is most sensitive to the day the control is ended, moderately sensitive to the day it is started, and only slightly sensitive to the length of the control. This is due to the timing of the end of the control determining the timing of the outbreak. We also see that continuing the control for another year has little impact on the magnitude of the divorce effect. (TIF) [file pcbi.1008292.s010.tif]

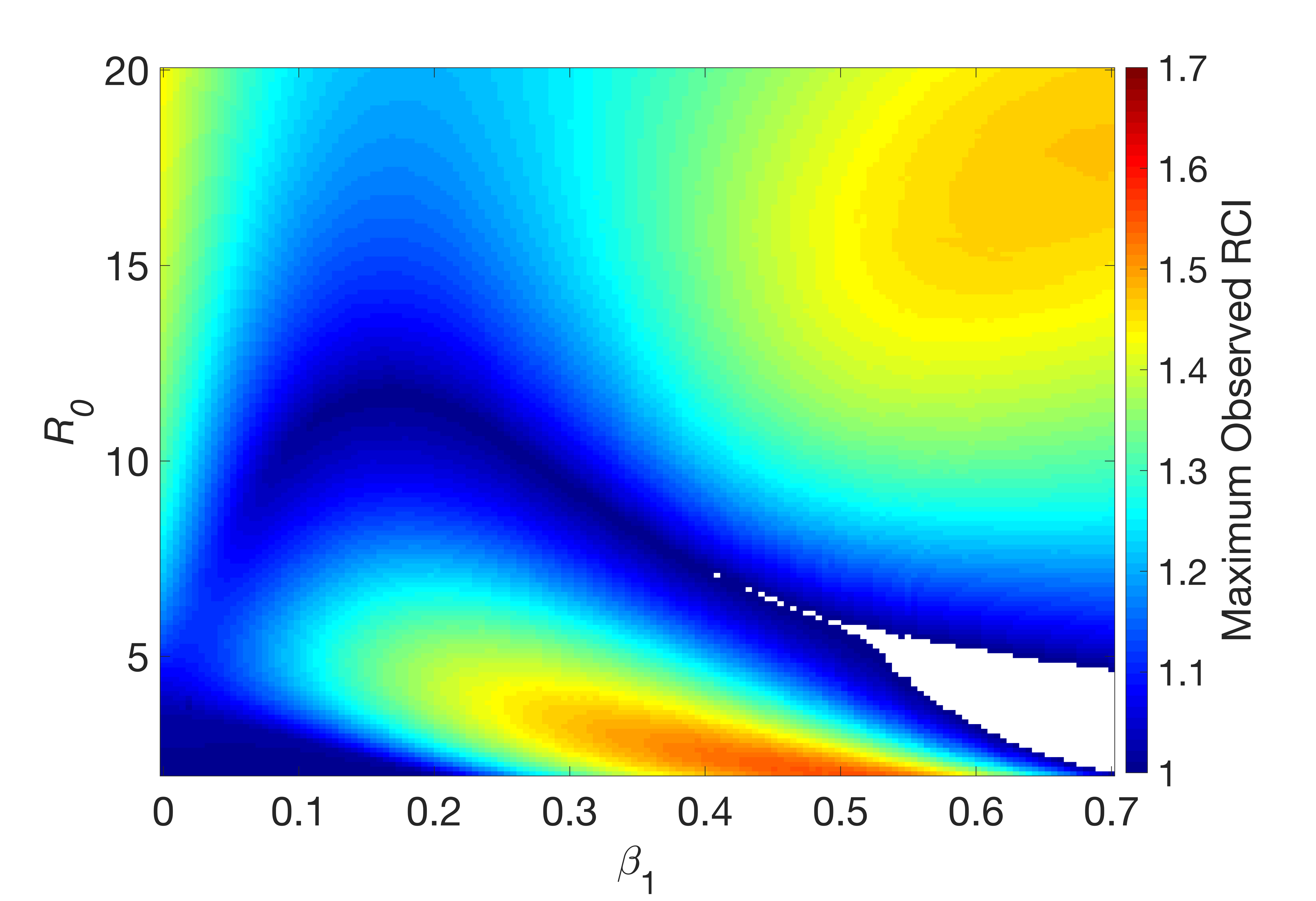

Supplement: S9 Fig — All controls are assumed to increase the vector mortality rate by 100% and to last for 1 year. The vector reproductive rate is assumed to have some average rate, r, and some level of seasonality (rs). The maximum RCI is found as the maximum observed RCI within 25 yrs after the end of a control. Here we see that the divorce effect is present throughout most of the parameter space. (TIF) [file pcbi.1008292.s011.tif]

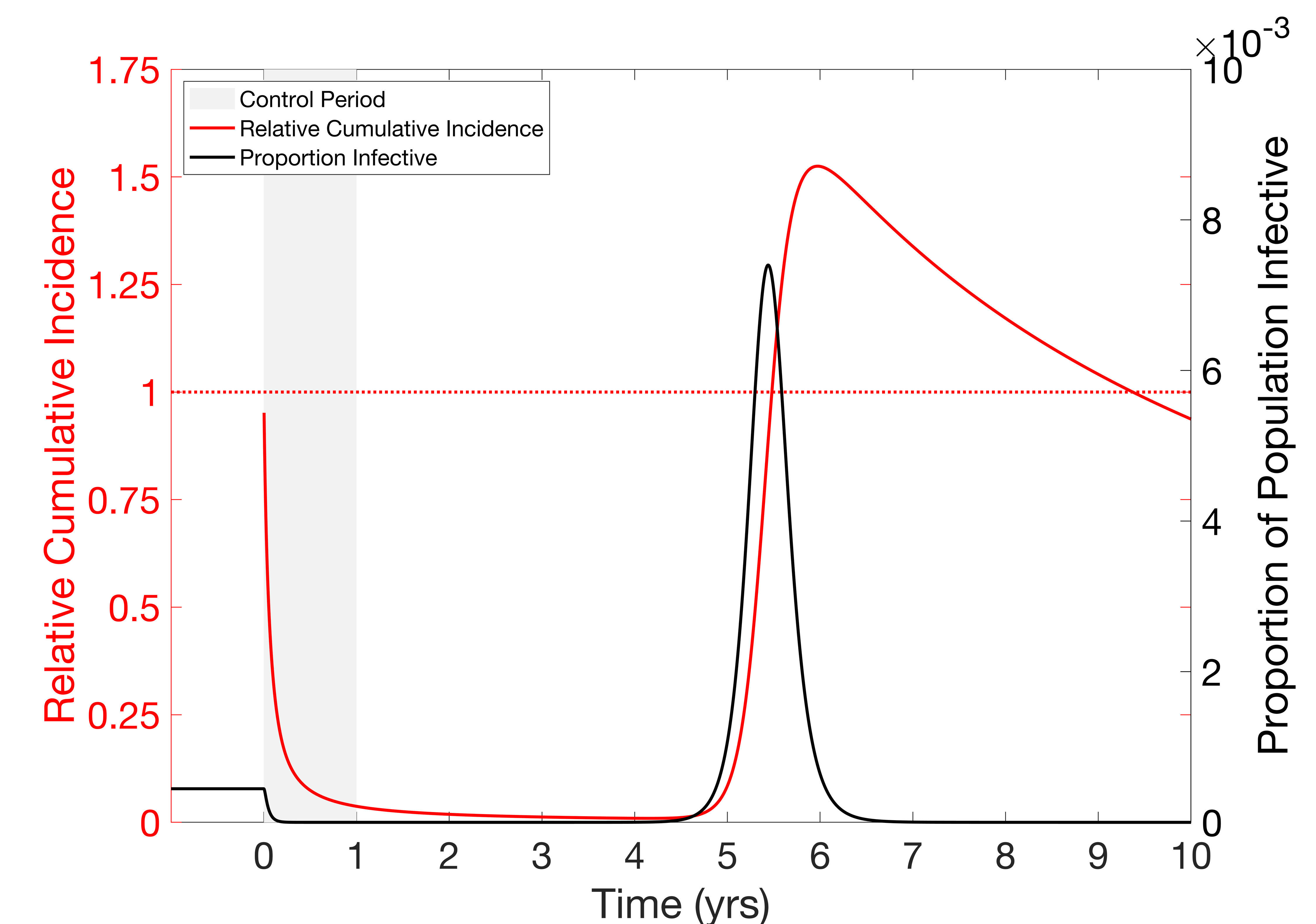

Supplement: S10 Fig — Figure shows the result of the non-seasonal host-vector model given in main text. Following a year of control, against an endemic infection (R0 = 5), in which the vector lifespan is reduced by 50% (δ = 73/year increased from δ = 36.5/year) incidence is reduced to near zero. After the control is stopped, we see a post-control outbreak in year 3, resulting in the divorce effect (peak RCI>1.5). (TIF) [file pcbi.1008292.s012.tif]

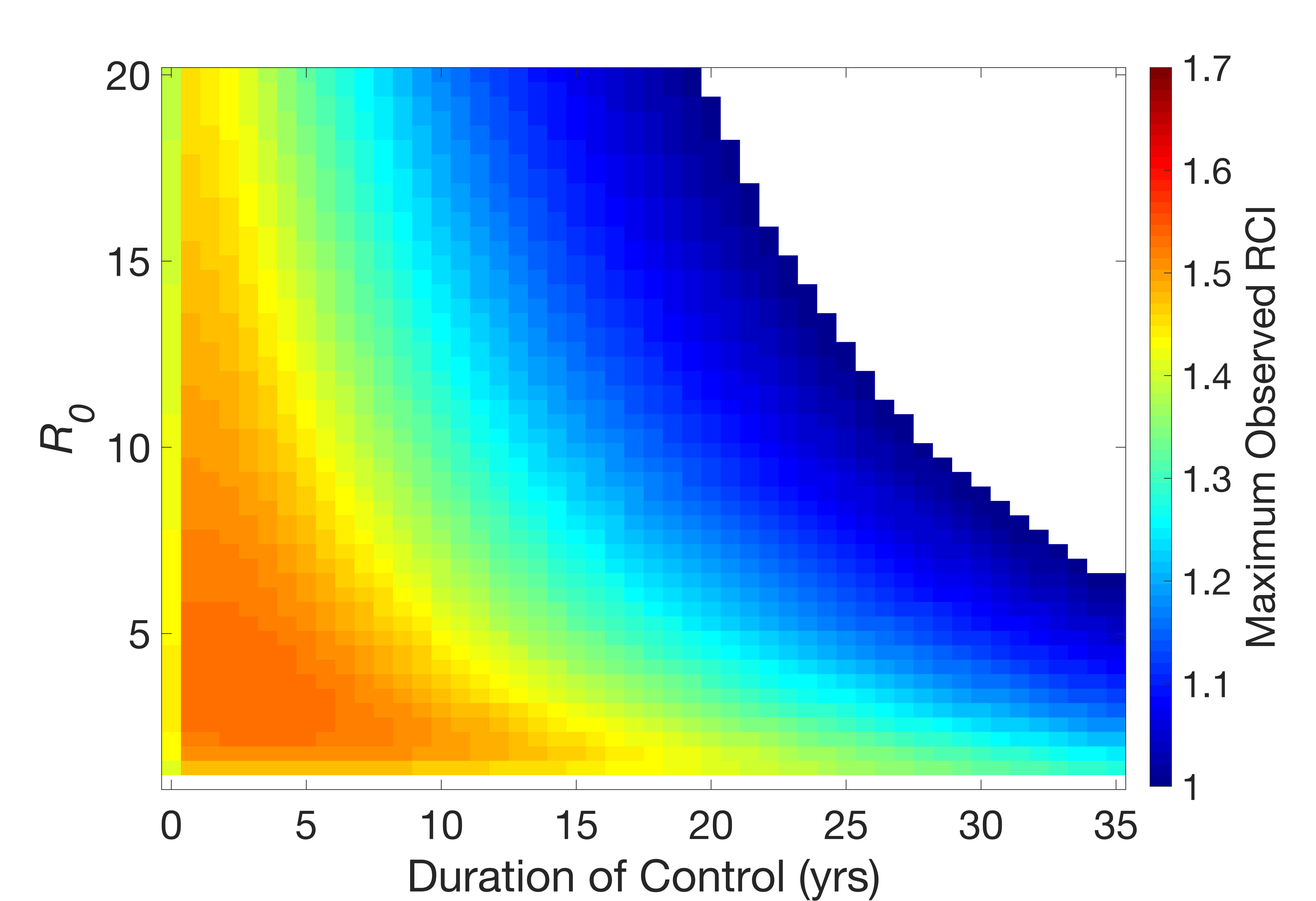

Supplement: S11 Fig — Maximum RCI is the highest RCI observed within 25 yrs following control of an infection with R0 = 5 beginning and ending on specified days. We see for the Host-Vector model that, much like the SIR model, the only way to avoid the divorce effect is to maintain control for more than 20 years. Parameters as given in the main text. Control decreases vector life-span by 50%. (TIF) [file pcbi.1008292.s013.tif]

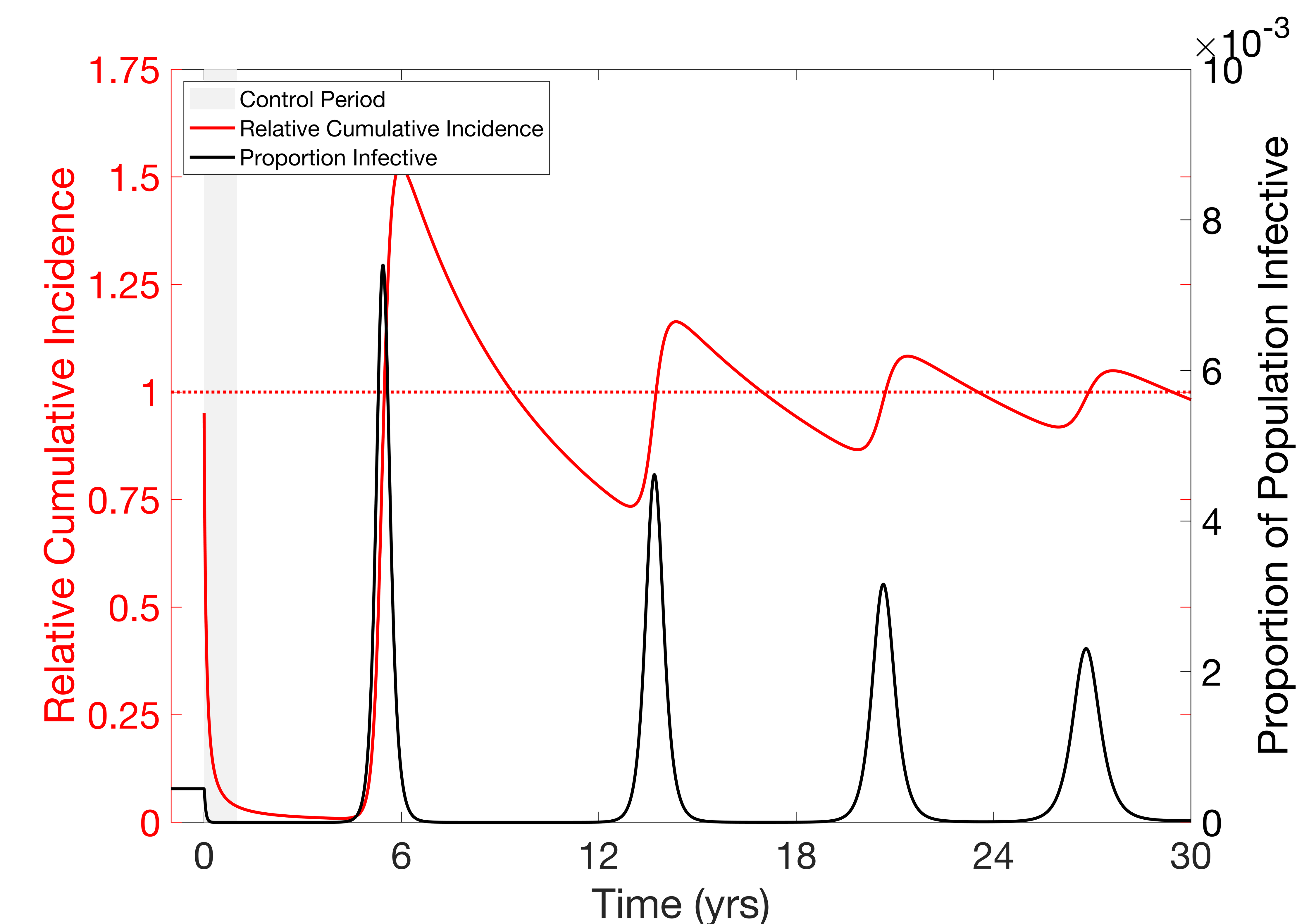

Supplement: S12 Fig — All parameters are as in Fig 3 of the main text. Following a one-year control, a large outbreak occurs in year 3, that brings RCI above 1. This outbreak deplenishes the susceptible population, resulting in no outbreaks for the next five years. Each subsequent outbreak is sufficiently large to bring RCI above 1. Around year 30, the system is still experiencing larger than normal outbreaks, bringing RCI slightly above 1. (TIF) [file pcbi.1008292.s014.tif]

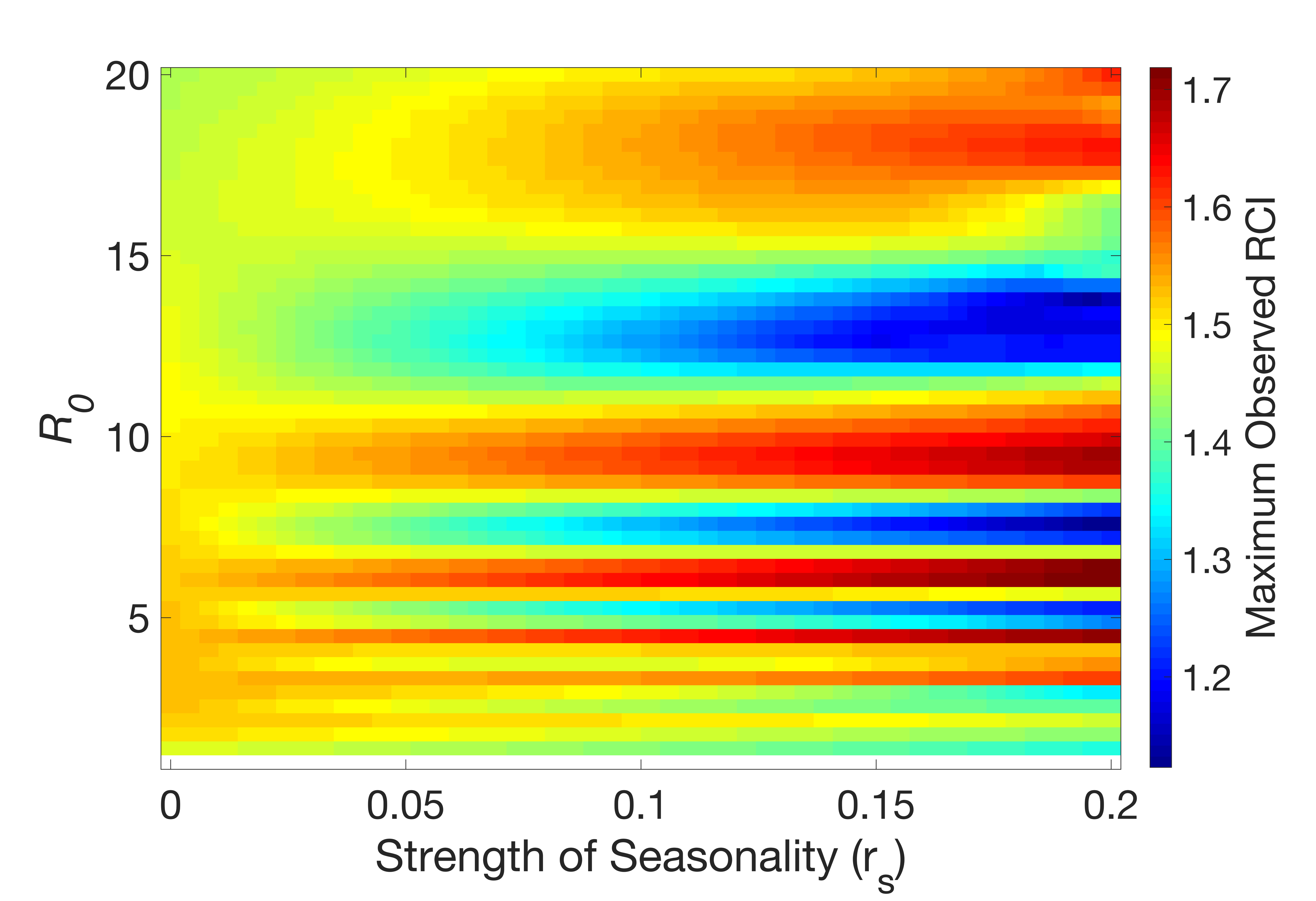

Supplement: S13 Fig — All controls are assumed to increase the vector mortality rate by 100% and to last for 1 year. The vector reproductive rate is assumed to have some average rate, r, and some level of seasonality (rs). The maximum RCI is found as the maximum observed RCI within 25 yrs after the end of a control. Here we see that the divorce effect is present throughout the parameter space. (TIF) [file pcbi.1008292.s015.tif]

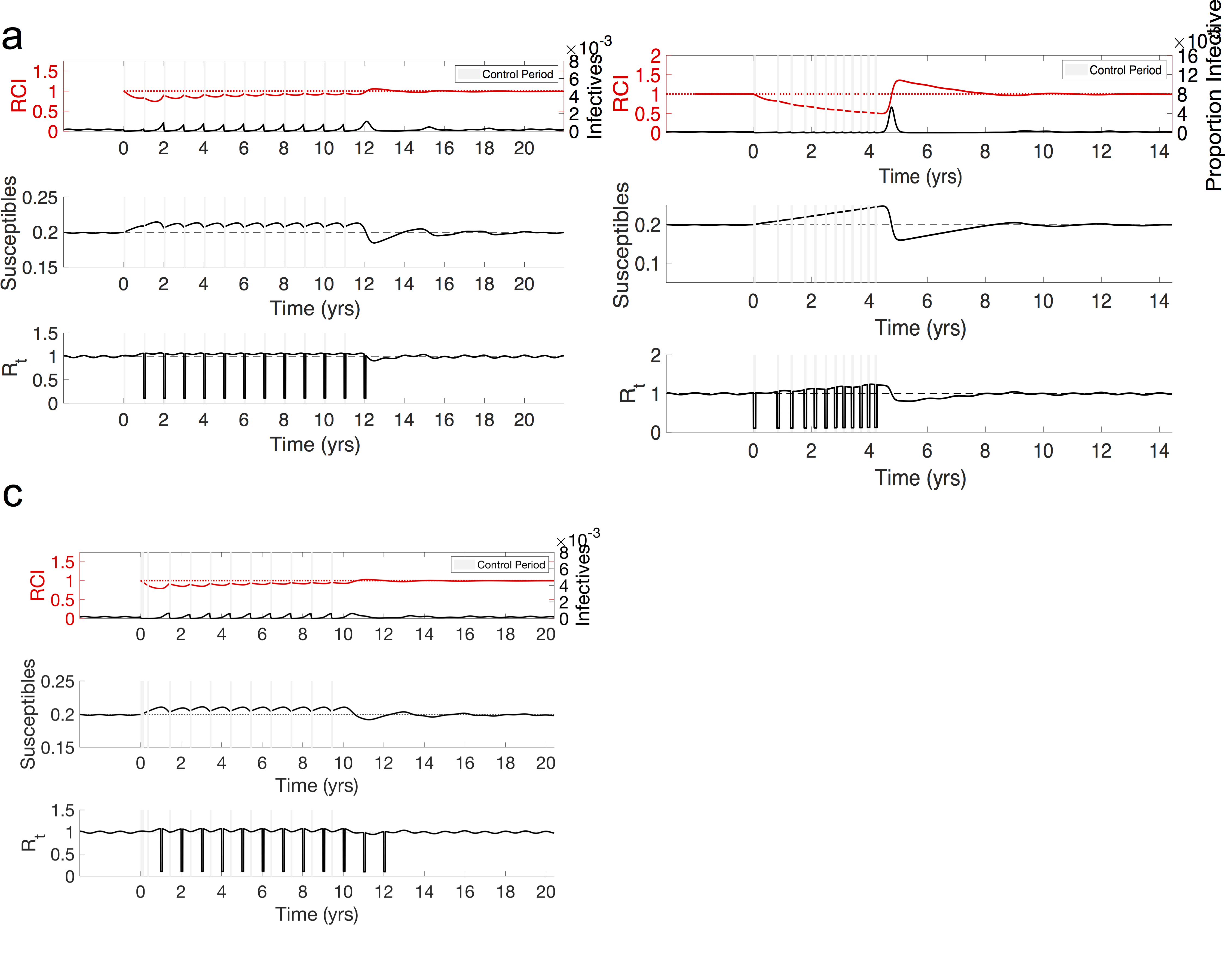

Supplement: S14 Fig — In the pulsed (a), reactive (b), and informed (c) techniques, we see the susceptible population begin growing with the first treatment and continue growing until the outbreak occurs after the 12th treatment, at which the susceptible population is quickly depleted. Likewise, the reproductive number, Rt, increases overall during this time with seasonal fluctuations, and reductions due to control periods. (TIF) [file pcbi.1008292.s016.tif]

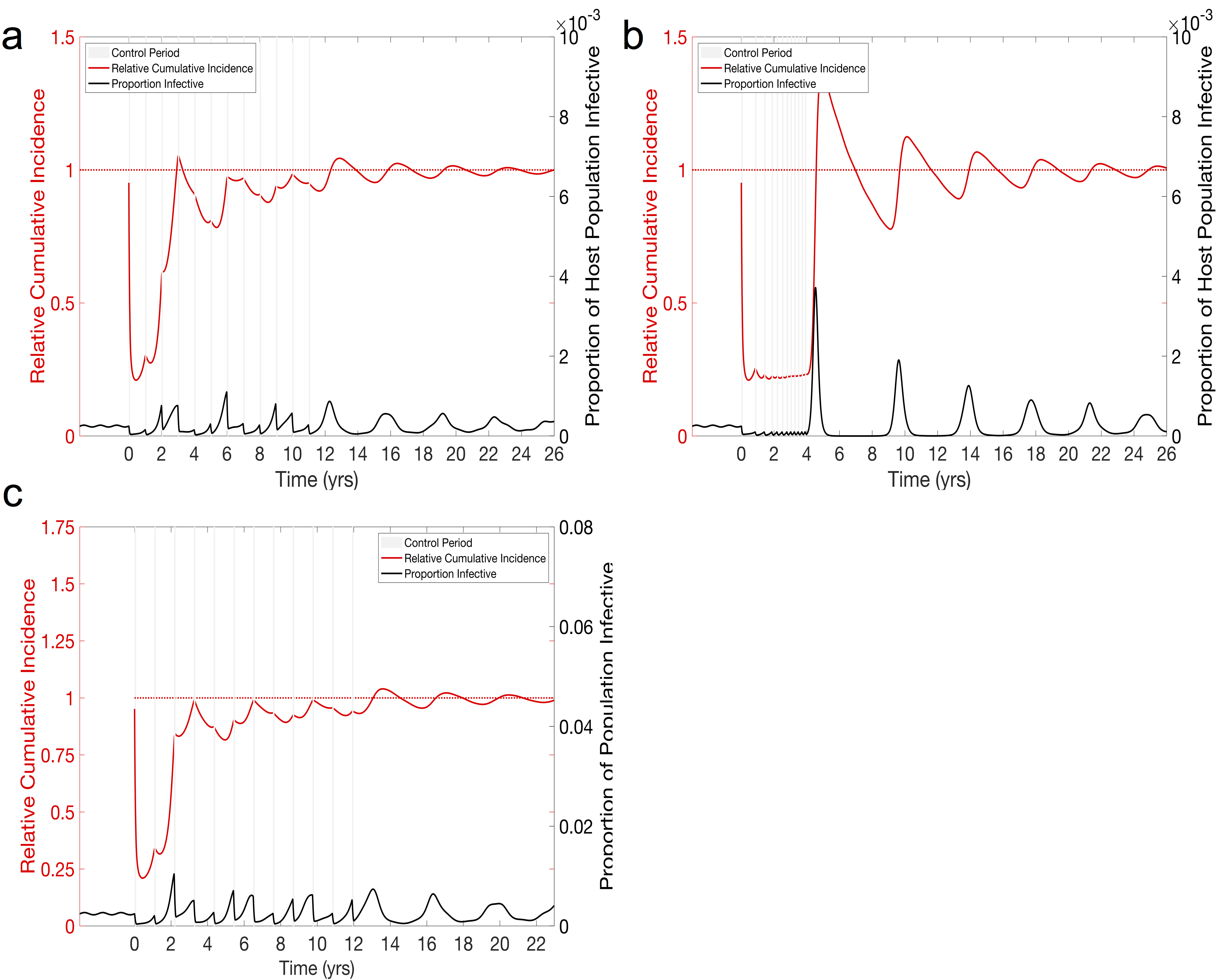

Supplement: S15 Fig — (a) Pulsed control for seasonal SIR model. Control (σ = 1) occurs yearly at a fixed time (when R0 is highest) for a fixed time (1 mo.). The control is effective at stopping the outbreak the first year, but seasonal outbreaks in subsequent years are of varied magnitudes due to the susceptible population being depleted in some years and replenished in others. An outbreak in year 2 is large enough to result in the divorce effect. Stopping the control program results in a large post-control outbreak and a divorce effect. (b) Reactive control for seasonal SIR. A fixed length (1 mo.) control is implemented once prevalence rises above a threshold (200 individuals in a population of 1 million). This stops the large outbreaks seen in the pulsed control, however the frequency of treatment increases as the susceptible population grows, and all treatments are depleted within the first four years. Stopping the control program results in a large outbreak and divorce effect. For all panels twelve 1 mo. controls are used to be consistent with the 1 yr. controls used in other figures. (c) Informed control in seasonal Host-vector model. Control works by increasing vector mortality by 100% for 1 month. The first control period occurs at time 0. The beginning of the next control period is decided at the end of the control period, and is the day that will result in the smallest divorce effect if control is stopped after that period (a maximum of 1 year between treatments). We see that this is capable of nearly eliminating the divorce effect, but there is only a negligible benefit to the control, with large yearly outbreaks. Importantly, this plan recommends waiting the full year, suggesting that the optimal timing of the next treatment may occur after this period. (TIF) [file pcbi.1008292.s017.tif]

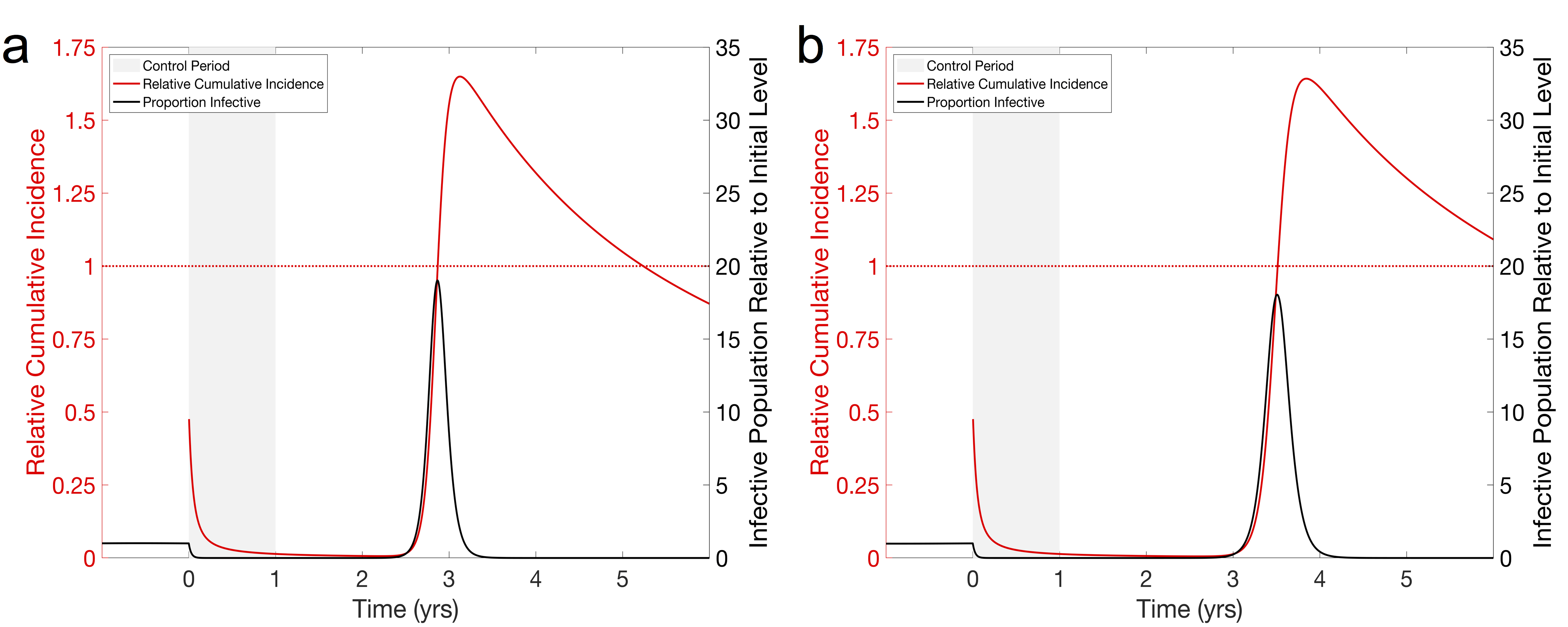

Supplement: S16 Fig — The population is (a) growing or (b) shrinking during the simulation at a rate of 25% per year. Beginning at time zero, a year-long 50% reduction in the transmission parameter of an endemic infection (R0 = 5) reduces prevalence of the infection to near zero for the length of the control, where it remains until time 2.5 yrs (a) or 3 yrs (b), at which point a large post-control outbreak occurs. RCI falls towards zero as prevalence remains low, but the post-control outbreak is large enough to bring RCI well above 1 (peak RCI = 1.67 and 1.64 for the growing and shrinking population, respectively). Note that, because of the changing population sizes within and between graphs, prevalence of infection is plotted on a relative scale on both graphs. (TIF) [file pcbi.1008292.s018.tif]

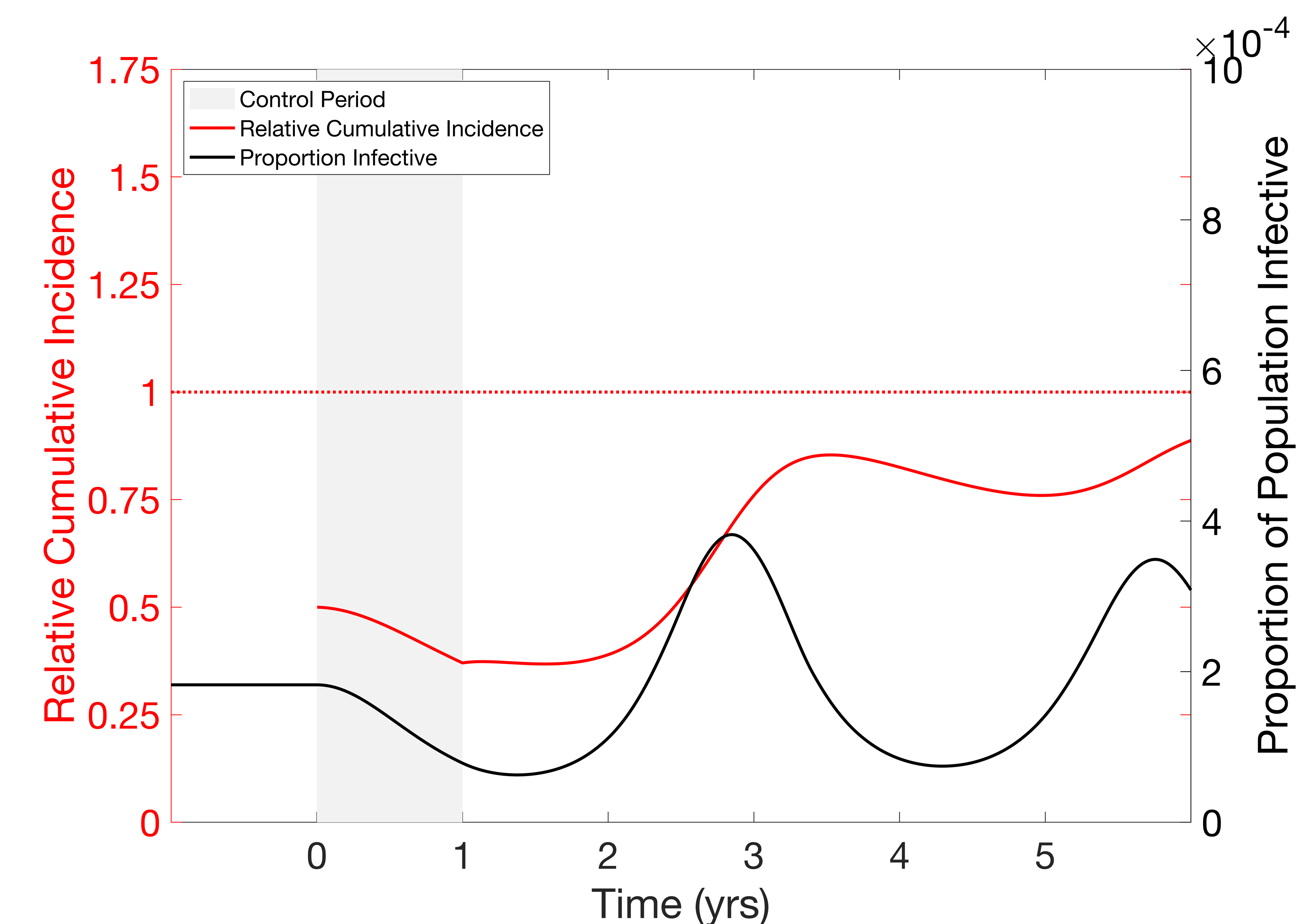

Supplement: S17 Fig — A vaccination program is put in place in which 50% of newborns are vaccinated for one year. All other parameters are as in Fig 1. The vaccination program is discontinued after the first year. While we see post-control outbreaks that bring incidence above the endemic level, they are not large enough to bring RCI above one, and RCI approaches 1 in the long run. (TIF) [file pcbi.1008292.s019.tif]

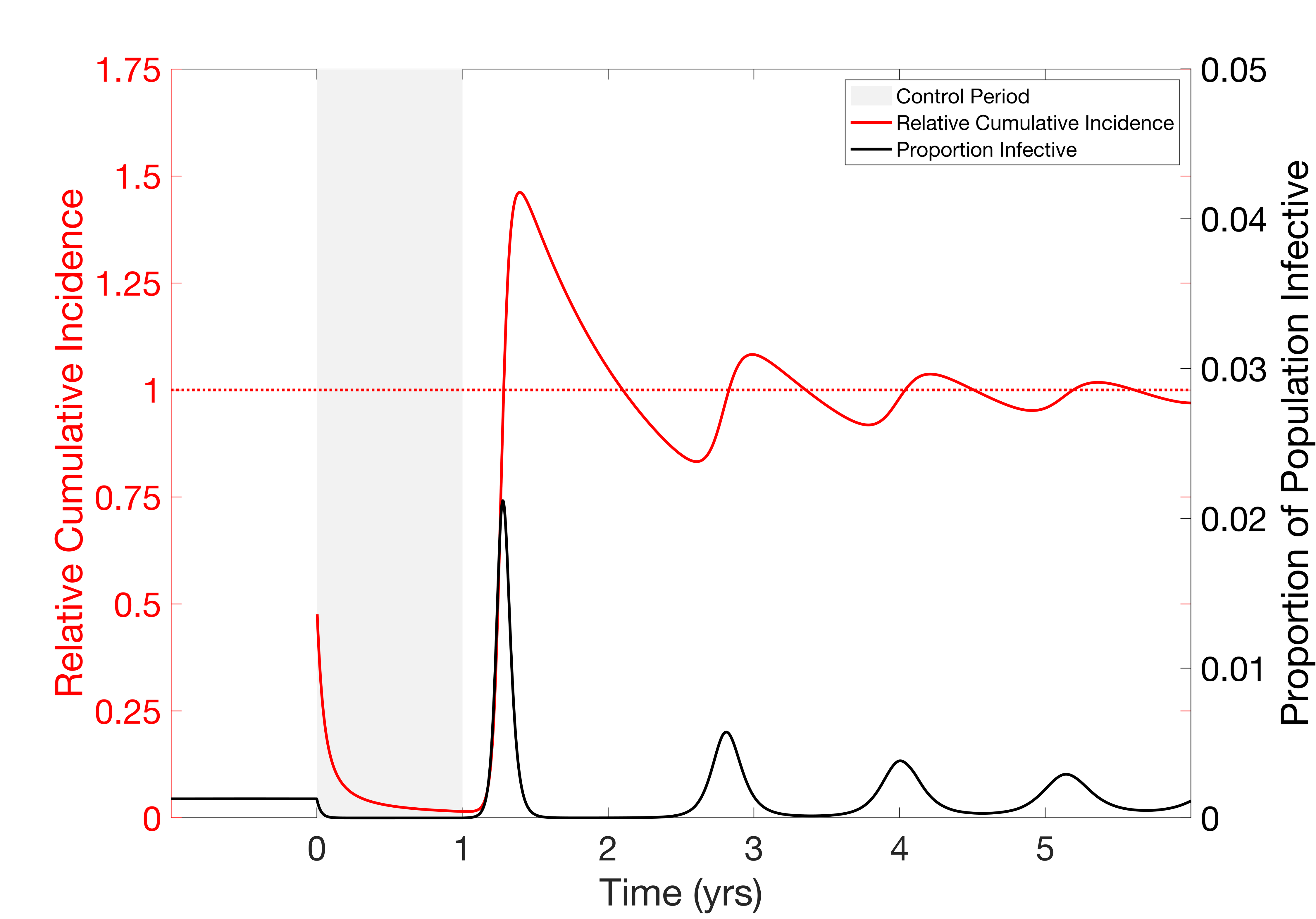

Supplement: S18 Fig — Beginning at time zero, a year-long 50% reduction in the transmission parameter of an endemic infection (R0 = 5) reduces prevalence of the infection to near zero for the length of the control, where it remains until time 1.5 yrs, at which point a large post-control outbreak occurs. RCI falls towards zero as prevalence remains low, but the post-control outbreak is large enough to bring RCI well above 1 (peak RCI is approx. 1.45). (TIF) [file pcbi.1008292.s020.tif]

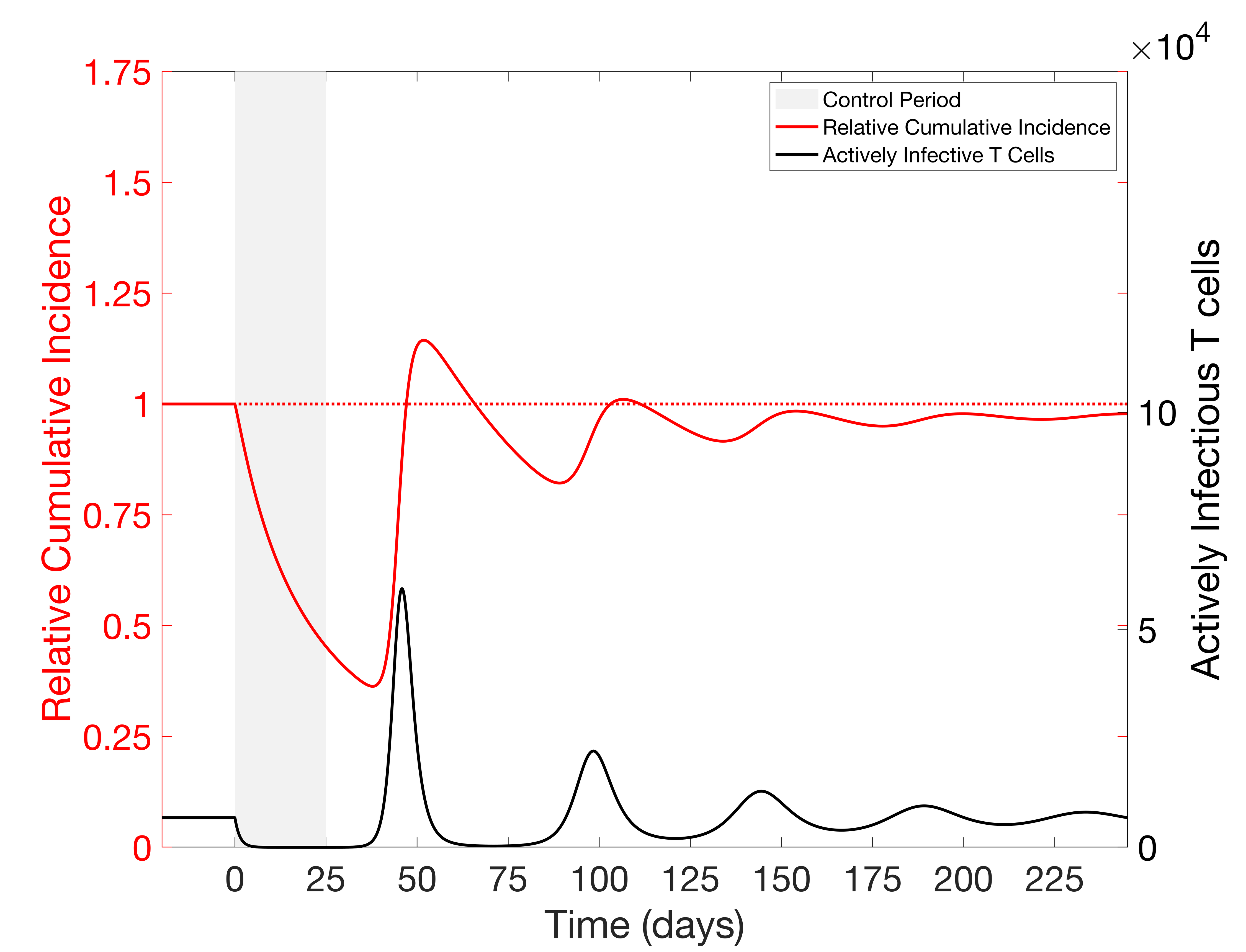

Supplement: S19 Fig — Beginning at time zero, a 25 day treatment occurs using a drug that combines a protease inhibitor and a reverse transcriptase inhibitor, both with 50% efficacy. This successfully reduces the infectious T cell count to near zero during and immediately following the treatment period. After the end of treatment, we see a transient increase in infectious T cells, bringing the relative cumulative incidence of T cell infection above one (max RCI»1.14). After years, RCI eventually approaches 1 from below. (TIF) [file pcbi.1008292.s021.tif]

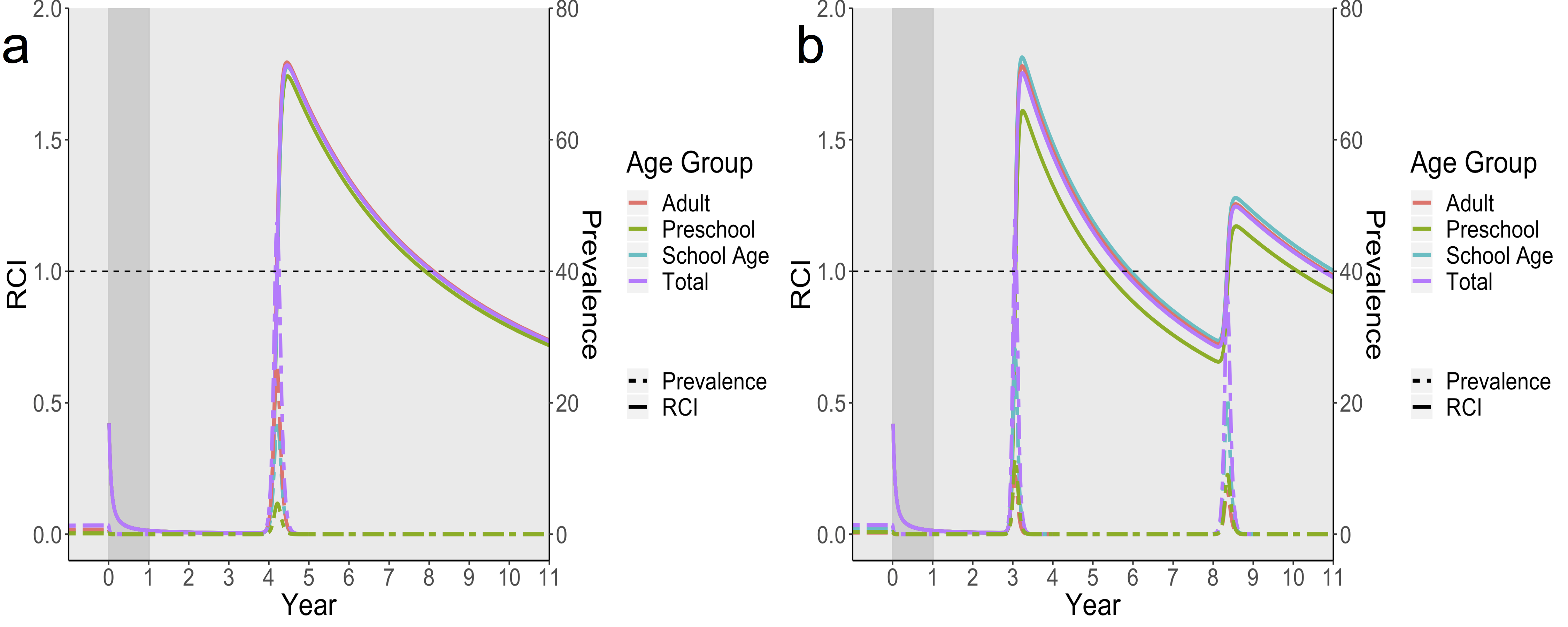

Supplement: S20 Fig — A control for a directly transmitted infection with R0 = 5 (a) and R0 = 15 (b) in a population of 9000 individuals is implemented at time 0, during which transmission is reduced by 50% for 1 year. At the end of the year, control is instantaneously removed. RCI quickly falls to 0 during the control period and remains there until a large outbreak in year 4 brings RCI up above 1 for all age groups. For the figure, prevalence, the number of individuals currently infective, (dashed lines) and RCI (solid lines) are shown for the total population, and age groups are shown aggregated into three groups: preschool (ages 0–5), school age (ages 6–18), and adult (ages 19+). (TIF) [file pcbi.1008292.s022.tif]

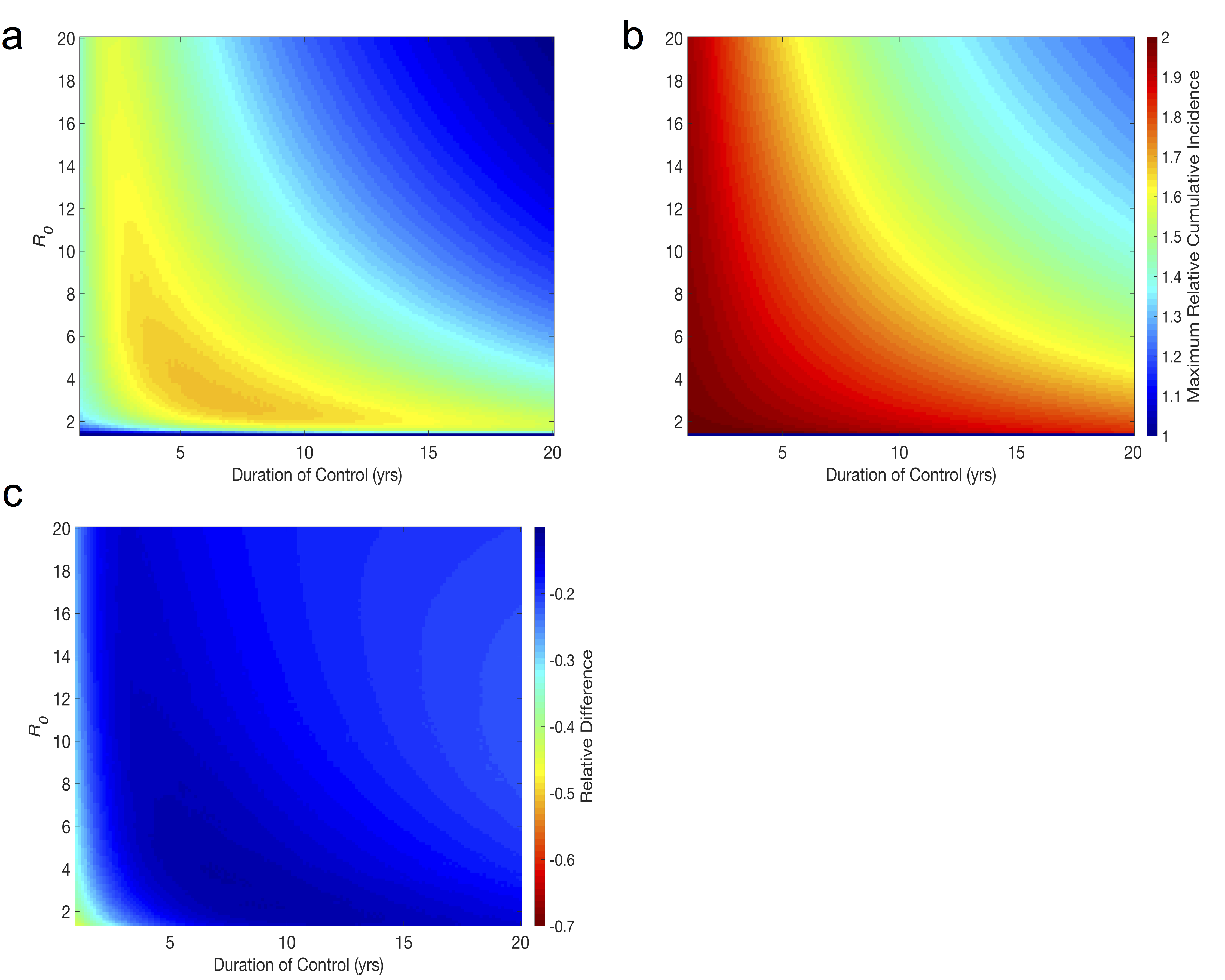

Supplement: S21 Fig — (a) Heat map of approximated and simulated magnitude of the divorce effect in terms of RCI. The analytical approximation predicts the divorce effect for all controls lasting less than 20 years, similar to what is observed in the SIR model (Fig 1(b)). However, it over estimates the observed maximum RCI throughout the parameter space, and does so drastically for a short control in a system with R0<3. (b) Relative difference between the observed and predicted maximum RCI. Calculated as (observed-predicted)/observed. The relative difference is small (< .25) throughout most of the parameter space except for short controls in systems with R0<3. (TIF) [file pcbi.1008292.s023.tif]

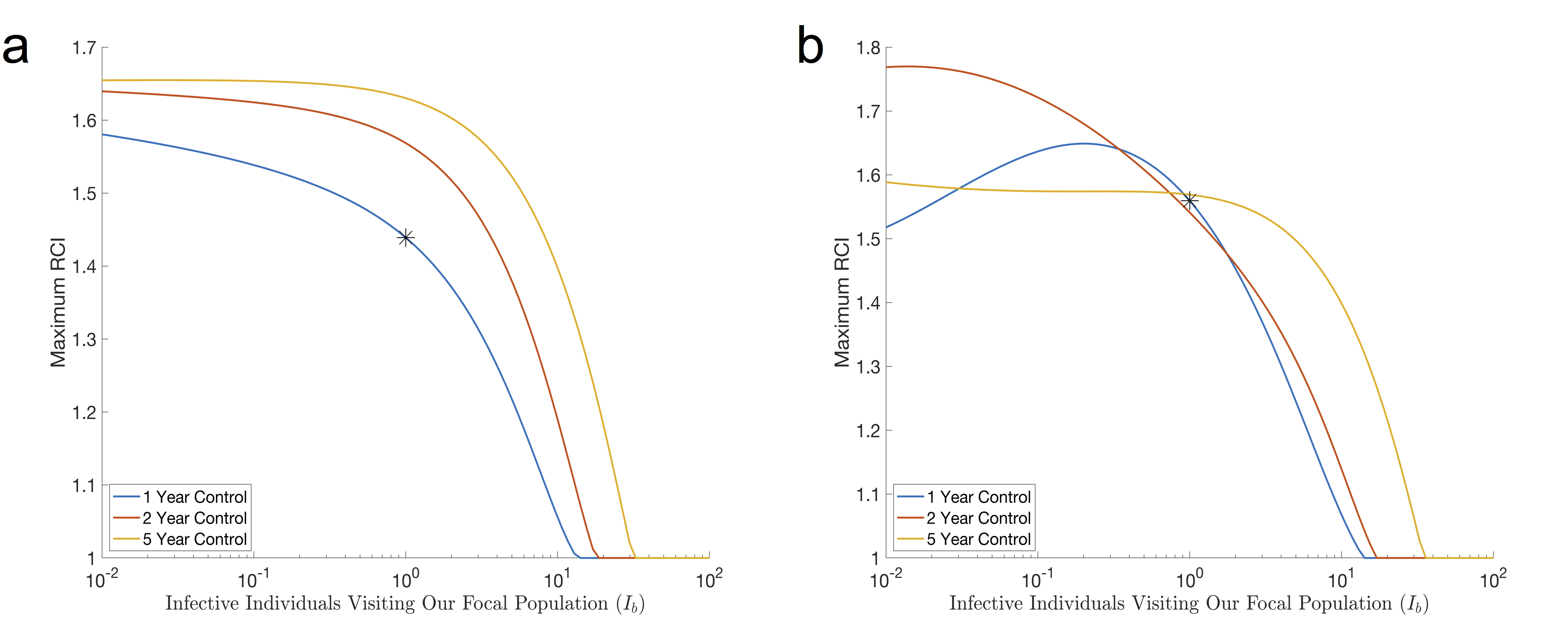

Supplement: S22 Fig — Sensitivity of the magnitude of the divorce effect to the background force of Infection in the (a) non-seasonal and (b) seasonal SIR models. Figure (a) is parameterized as in Fig 1(a) (R0 = 5) and (b) is parameterized as in Fig 2(a) (R0 = 5, β1 = .02). All controls are assumed to last one, two, or five years, beginning at t = 0. We see that for a sufficiently high number of infective individuals visiting our focal population (Ib), the divorce effect is eliminated. We choose a seemingly realistic value of Ib = 1 (stars) for our models, compared to an endemic level of 183 infective individuals for these parameter values in the nonseasonal model. This value will keep the number of infectives from falling to arbitrarily small values while not eliminating the divorce effect. We note that values of Ib that are sufficiently large to eliminate the divorce effect would require Ib to be roughly the same size as the endemic infection level. (TIF) [file pcbi.1008292.s024.tif]

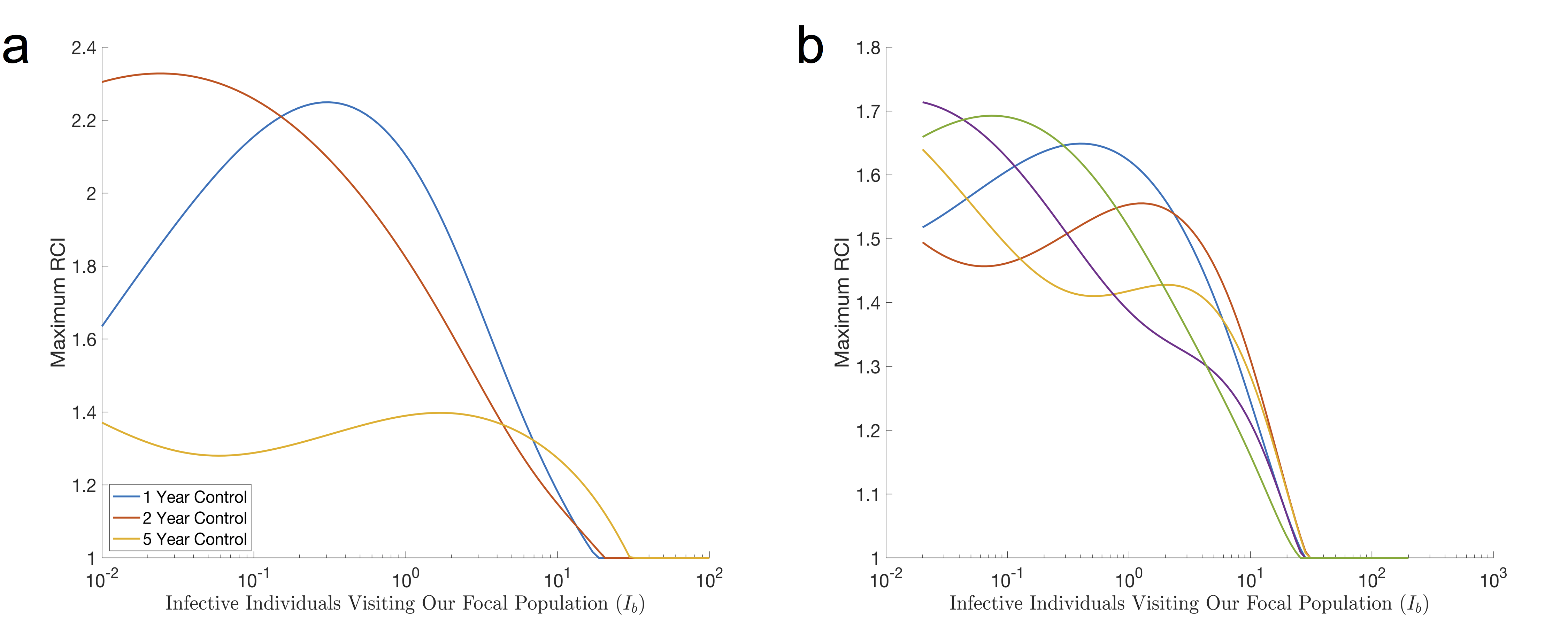

Supplement: S23 Fig — (a) Increasing seasonality (β1 = .1 in contrast to β1 = .02 in S22 Fig) increases the variation in the divorce effect seen at differing background force of infections. (b) Likewise, the timing of the start of the control (days 0, 73, 146, 219, 292) has a significant effect on the magnitude of the divorce effect seen at a particular background force of infection. (TIF) [file pcbi.1008292.s025.tif]
