# Supplementary material for: After the honeymoon, the divorce: Unexpected outcomes of disease control measures against endemic infections
Source: PLoS Comput Biol. 2020 Oct 19;16(10):e1008292. doi: 10.1371/journal.pcbi.1008292 (PMC7595641; doi:10.1371/journal.pcbi.1008292)
Supplement: S1 Table — (DOCX) [file pcbi.1008292.s026.docx]

| **Symbol** | **Parameter** | **Value** |
| --- | --- | --- |
| $\boldsymbol{\lambda}$ | Susceptible Cell Recruitment Rate | ${10}^{-4}\text{ }\text{mL}^{-1}\text{day}^{-1}$ |
| $\boldsymbol{d}_{\boldsymbol{T}}$ | Susceptible Cell Mortality Rate | $.01\text{ day}^{-1}$ |
| $\boldsymbol{\alpha}_{\boldsymbol{L}}$ | Fraction of Infections Resulting in Latency | .001 |
| $\boldsymbol{k}$ | Infection Rate Constant | $2.4\text{x}{10}^{-8}\text{mL }\text{day}^{-1}$ |
| $\boldsymbol{d}_{\boldsymbol{L}}$ | Death Rate of Latent Cells | $.004\text{ day}^{-1}$ |
| $\boldsymbol{\alpha}$ | Latent Cell Activation Rate | $.1\text{ }\text{day}^{-1}$ |
| $\boldsymbol{\delta}$ | Death Rate of Actively Infected Cells | $1\text{ }\text{day}^{-1}$ |
| $\boldsymbol{c}$ | Free Virus Clearance Rate | $23\text{ }\text{day}^{-1}$ |
| $\boldsymbol{N}$ | Burst Size | 3000 |
| $\boldsymbol{\epsilon}_{\boldsymbol{RT}}$ | Efficacy of Reverse Transcriptase inhibitors | .5 |
| $\boldsymbol{\epsilon}_{\boldsymbol{PI}}$ | Efficacy of Protease Inhibitors | .5 |
